# Discovery and Characterization of ZUFSP/ZUP1, a Distinct Deubiquitinase Class Important for Genome Stability

**DOI:** 10.1016/j.molcel.2018.02.023

**Published:** 2018-04-05

**Authors:** Dominika Kwasna, Syed Arif Abdul Rehman, Jayaprakash Natarajan, Stephen Matthews, Ross Madden, Virginia De Cesare, Simone Weidlich, Satpal Virdee, Ivan Ahel, Ian Gibbs-Seymour, Yogesh Kulathu

**Affiliations:** 1 MRC Protein Phosphorylation & Ubiquitylation Unit, School of Life Sciences, University of Dundee, Dow Street, Dundee DD1 5EH, UK; 2 DNA Damage Response Laboratory, Sir William Dunn School of Pathology, South Parks Road, Oxford OX1 3RE, UK

**Keywords:** deubiquitinating enzyme, DUB, uniquitin binding domain, DNA repair, DNA damage response, ubiquitin signaling, polyubiquitin, Lys63 chains

## Abstract

Deubiquitinating enzymes (DUBs) are important regulators of ubiquitin signaling. Here, we report the discovery of deubiquitinating activity in ZUFSP/C6orf113. High-resolution crystal structures of ZUFSP in complex with ubiquitin reveal several distinctive features of ubiquitin recognition and catalysis. Our analyses reveal that ZUFSP is a novel DUB with no homology to any known DUBs, leading us to classify ZUFSP as the seventh DUB family. Intriguingly, the minimal catalytic domain does not cleave polyubiquitin. We identify two ubiquitin binding domains in ZUFSP: a ZHA (ZUFSP helical arm) that binds to the distal ubiquitin and an atypical UBZ domain in ZUFSP that binds to polyubiquitin. Importantly, both domains are essential for ZUFSP to selectively cleave K63-linked polyubiquitin. We show that ZUFSP localizes to DNA lesions, where it plays an important role in genome stability pathways, functioning to prevent spontaneous DNA damage and also promote cellular survival in response to exogenous DNA damage.

## Highlights

• DUB profiling identifies ZUFSP/ZUP1 as distinct DUB class that cleaves K63 chains
• High-resolution crystal structure reveals mechanisms of Ub recognition and catalysis
• Ub binding UBZ and ZHA domains identified in ZUFSP are essential for DUB activity
• ZUFSP prevents genome instability and promotes cellular survival following DNA damage

## Graphical Abstract

**Figure:**
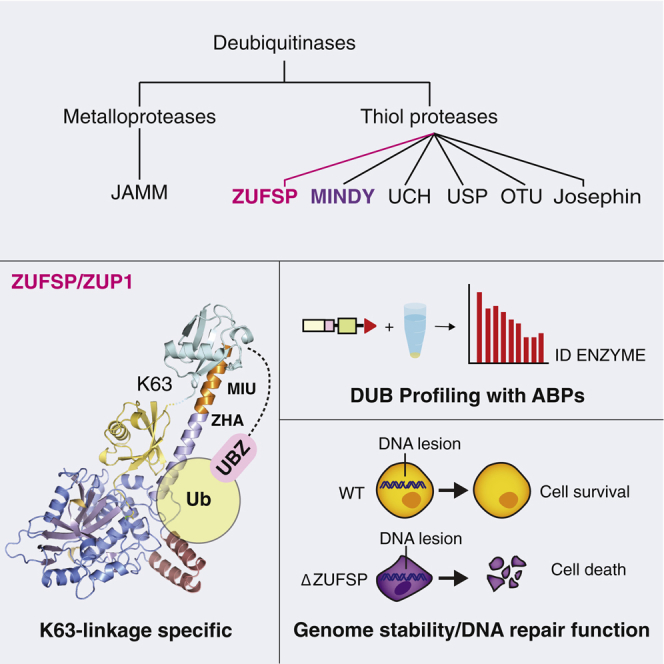

## Teaser

Kwasna et al. discover ZUFSP/ZUP1 as a new deubiquitinase class specific at cleaving K63-linked polyubiquitin. They identify two ubiquitin binding domains in ZUFSP that are essential for enzyme activity. ZUFSP is a predominantly nuclear DUB with important roles in maintaining genome stability.

## Introduction

The posttranslational modification (PTM) of proteins by ubiquitin (Ub) is a signal that is used in a broad spectrum of cellular processes (Kulathu and Komander, 2012). The attachment of Ub to a substrate protein typically occurs via the formation of an isopeptide bond that links the C-terminal carboxy group of Ub with the ε-amino group of a lysine residue in the substrate (Hershko and Ciechanover, 1998, Pickart, 2001). Ub itself contains seven lysine residues (K6, K11, K27, K29, K33, K48, K63) and an N-terminal methionine (M1) that can serve as attachment sites for another Ub resulting in the formation of Ub chains (polyUb). Thus, lysines on proteins can be modified with a single Ub, monoubiquitylation, or with polyUb of different linkage types. Importantly, polyUb of different linkage types signal different functional outcomes, thus making ubiquitylation a versatile PTM (Yau and Rape, 2016).

To function as a signal, the different Ub modifications have to be decoded by proteins with Ub binding domains (UBDs). There are several different types of UBDs known so far and they employ different modes of binding to recognize Ub (Husnjak and Dikic, 2012). Intriguingly, many of the UBDs are α-helical in nature and most interact with the hydrophobic Ile44 patch (comprising L8, I44, H68, and V70) on Ub (Kulathu and Komander, 2012). Most UBDs bind to monoUb with weak affinity, with K_d_ typically greater than 100 μM (Hurley et al., 2006). However, many proteins contain tandem-binding domains or utilize other forms of cooperativity to achieve high-affinity interactions essential for different Ub signals to be selectively recognized in a physiological setting (Husnjak and Dikic, 2012).

As it is used as a signal in so many different cellular processes, it is important for ubiquitylation to be tightly regulated. Indeed, ubiquitylation is a dynamic modification that is reversed by dedicated proteases called deubiquitinases, or DUBs. There are approximately 100 human DUBs that until recently were classified into five distinct families (Clague et al., 2013, Ronau et al., 2016). Recently, a sixth family of DUBs (MINDY family) was discovered (Abdul Rehman et al., 2016), and these MINDY DUBs show exquisite selectivity in cleaving K48-linked polyUb. Such linkage selectivity is present in few other DUBs, whereas the vast majority of DUBs are non-selective and will cleave all types of polyUb (Faesen et al., 2011, Mevissen et al., 2013). Linkage selective DUBs cleave within chains to trim the polyUb and can thereby edit the Ub signal (Komander et al., 2009). Such DUBs therefore rely on recognition of both distal Ub (S1) and proximal Ub (S1′) of a diUb positioned across the catalytic site to achieve specificity of cleavage. Being important regulators of the Ub system, DUBs are increasingly implicated in human disease, and, as a result, there has been considerable interest in recent years in exploring DUBs as drug targets (Leznicki and Kulathu, 2017).

One cellular process where ubiquitylation plays important roles is the DNA damage response (DDR), a cellular signal transduction pathway that functions to maintain the integrity of DNA (Jackson and Durocher, 2013). Ubiquitylation has emerged as a key regulatory mechanism to orchestrate protein signaling within the DDR, with arguably the best-characterized DDR pathway being the cellular response to double-strand breaks (DSBs). Ub signaling during DSB repair is driven by the E3 Ub ligases RNF8 and RNF168, which mainly catalyze K63-linked polyUb on histones and possibly other chromatin-associated proteins (Schwertman et al., 2016). In addition to histones, ubiquitylation of specific protein targets is often associated with activation of particular DDR pathways. For example, ubiquitylation of the DNA replication sliding clamp PCNA and the single-stranded binding protein RPA occurs in response to DNA replication stress (García-Rodríguez et al., 2016). Functionally, RPA ubiquitylation has been suggested to promote homologous recombination (HR) at stalled forks, which may be due to inappropriate retention of RPA on single-stranded DNA, thereby inhibiting downstream RAD51 loading and HR (Feeney et al., 2017, Inano et al., 2017). Collectively, the above examples illustrate the multifaceted manner by which Ub-dependent signaling mechanisms function to promote the DDR and maintain genome stability. Importantly, with the widespread roles for ubiquitylation in the DDR, this process is also tightly regulated by DUBs, many of which have been reported to regulate various genome maintenance pathways (Kee and Huang, 2015).

Here, we expand our understanding of DUBs and the regulation of Ub signaling in DNA repair with the discovery of ZUFSP as a DUB that is highly selective at cleaving K63-linked polyUb. A high-resolution crystal structure of ZUFSP in complex with Ub reveals that the catalytic domain uses a unique Ub binding motif to bind to the distal Ub. Further, the architecture of the catalytic domain is unique among DUBs. Based on these observations, we classify ZUFSP as the seventh family of human DUBs. Interestingly, ZUFSP binds to several proteins involved in DNA replication and repair and indeed detailed analyses reveal ZUFSP as a regulator of genome stability.

## Results

### Activity-Based Profiling Identifies ZUFSP as a Putative DUB

**Figure 1:**
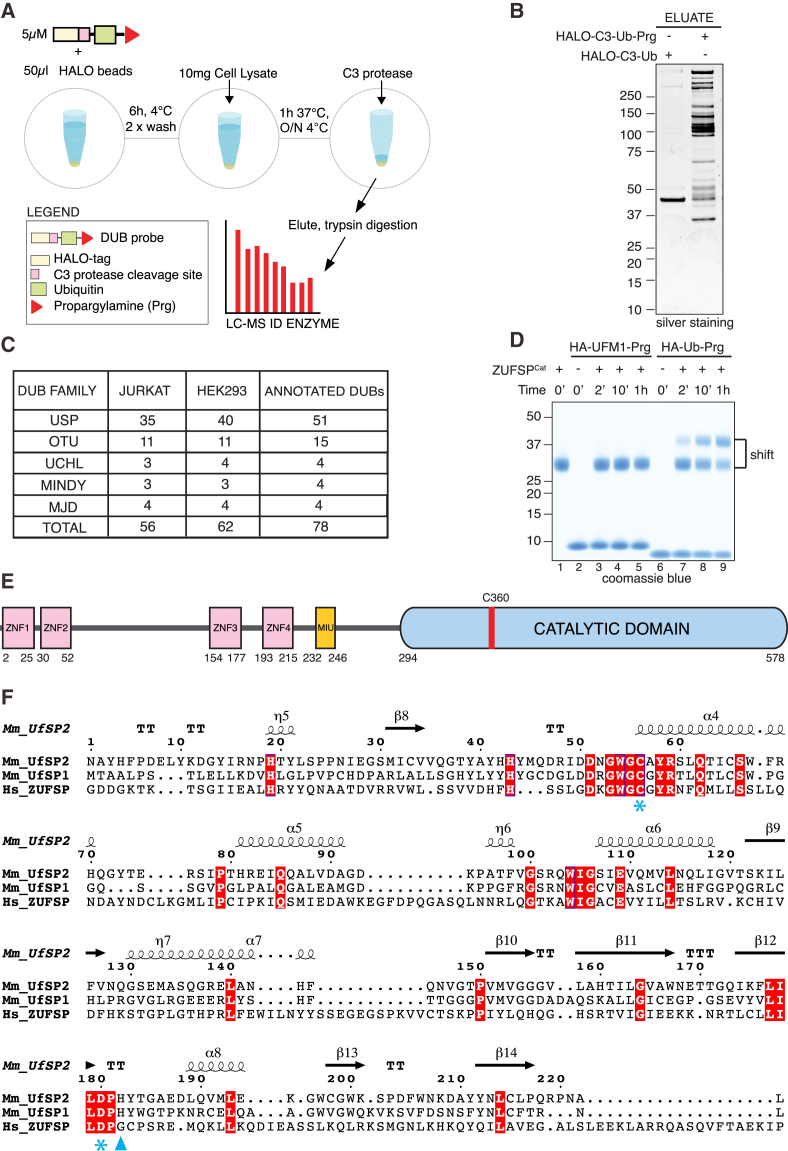
Identification of ZUFSP as a DUB (A) Schematic representation of DUB profiling pipeline. (B) Halo-tagged Ub or propargylated Ub (Ub-Prg) coupled to HaloLink resin was incubated with cell extracts. Captured material was separated by SDS-PAGE and silver stained. (C) Summary of DUBs identified by mass spectrometry using the Halo-C3-Ub-Prg probe with lysates of HEK293 and Jurkat cells. The last column shows the total number of annotated DUBs in each family (metalloproteases are not included). (D) The ZUFSP minimal catalytic domain (294–578) was reacted with the HA-Ub-Prg or HA-UFM1-Prg probes for indicated times. Reaction products were separated by SDS-PAGE gel stained using Coomassie blue. (E) Schematic domain structure of ZUFSP. Zinc finger (ZNF) domains (pink), motif interacting with Ub (MIU) domain (orange), and minimal catalytic domain (blue) are depicted. (F) Sequence alignment of human ZUFSP with mouse (Mm) Ufsp1 and Ufsp2. Secondary structure elements are shown for Ufsp2. Conserved catalytic residues are highlighted with blue asterisks; missing His is highlighted with a blue triangle. Fully conserved residues are shaded in red. See also Figure S1.

Since our recent discovery of a new family of DUBs (Abdul Rehman et al., 2016), we sought to determine whether there were other yet-to-be identified DUBs. Ub-based suicide probes have been instrumental in the identification of several DUBs (Hewings et al., 2017). We therefore established a sensitive and robust pipeline using chemical probes in combination with proteomic approaches to isolate and identify DUBs from cell extracts (Figures 1A and 1B). Using propargylated Ub (Ub-Prg), a potent and selective modifier of DUBs (Ekkebus et al., 2013), we set up an optimized pipeline to maximize recovery of DUBs, with which we capture the majority of cysteine-based DUBs (Figures 1C and S1A–S1C). When performed with lysates from two different cell lines, we captured a unique set of DUBs from each cell line. The robustness of the pipeline established here is also highlighted by the presence of MINDY DUBs, which had previously not been identified using Ub-based suicide probes. Intriguingly, we repeatedly observed the presence of ZUFSP (Zinc finger with UFM1-specific peptidase domain protein) in our pull-downs (Figure S1D). UFM1 is an important Ub-like modifier whose functional roles are still poorly understood (Daniel and Liebau, 2014). However, ZUFSP is thought to be an inactive UFM1 peptidase as it lacks a key catalytic residue (Figure 1F). To rule out that ZUFSP was a contaminant in our Ub suicide probe pull-downs, we expressed and purified the catalytic domain of ZUFSP and monitored its activity toward Ub-Prg. Indeed, the predicted catalytic domain of ZUFSP is readily modified by Ub-Prg (Figures 1D and 1E). In contrast, ZUFSP is not modified by UFM1-Prg suggesting that ZUFSP may be a Ub-specific protease (Figures 1D and S1E). A sequence alignment of ZUFSP with the other known Ufm1 proteases Ufsp1 and Ufsp2, however, reveals that the His residue that is part of the catalytic triad of both Ufsp1 and Ufsp2 is missing in ZUFSP (Figure 1F). This raised the question of whether and how ZUFSP could be an active DUB.

### ZUFSP Forms a Separate DUB Class

To understand the molecular details of Ub recognition and catalysis by this putative DUB, we aimed to determine the crystal structure of ZUFSP. Human ZUFSP is a modular protein comprising 578 amino acids (aa) with four C2H2 type zinc fingers at the N terminus followed by a conserved motif interacting with Ub (MIU) juxtaposed to the catalytic domain (residues 294–578) at the C terminus (Figure 1E). However, this minimal catalytic domain was not stable and resisted crystallization. We recently showed that the activity of MINDY1 is greater in the presence of its Ub binding MIU motifs (Abdul Rehman et al., 2016). We therefore decided to expand the ZUFSP construct boundaries to include the conserved MIU motif, which we hypothesized may be involved in Ub recognition and catalysis. This longer construct (residues 232–578), ZUFSP^MIU-Cat^, expresses at higher levels and has improved solubility. ZUFSP^MIU-Cat^ was complexed with Ub-Prg and crystals of the complex were obtained at 14 mg/mL. The structure was determined by MAD phasing using selenomethionine substituted ZUFSP and refined to the values shown in Table 1. This 1.7-Å resolution crystal structure reveals several unique features about ZUFSP and its unique mode of distal Ub recognition.

**Table 1:** Data Collection and Refinement Statistics

	Peak	Inflection	Native
**Data collection**			

Beamline	ID29, ERSF	ID29, ESRF	I04, DLS
Wavelength (Å)	0.97264	0.97923	0.91587
Space group	P6_5_22	P6_5_22	P6_5_22
Total reflections	4,119,930	4,562,263	1,295,453

**Cell dimensions**			

a, b, c (Å)	84.64, 84.64, 201.74	84.45, 84.45, 201.32	84.48, 84.48, 201.87
α, β, γ (°)	90.00, 90.00, 120.00	90.00, 90.00, 120.00	90.00, 90.00, 120.00
Resolution (Å)	68.89–1.89	49.45–1.89	100.93–1.74
R_merge_	0.293 (3.467)	0.149 (3.681)	0.082 (0.99)
R_meas_	0.296 (3.504)	0.151 (3.709)	0.085 (1.022)
I/σ(I)	15.6 (2.1)	34.9 (2.6)	25.8 (4.0)
Completeness (%)	100 (100)^a^	100 (100)^a^	100.0 (100.0)
Multiplicity	62.3 (46.3)^a^	65.7 (67.2)^a^	29.0 (30.6)
CC1/2	0.99(0.76)	1.0 (0.83)	1.0 (0.93)

**Refinement**			

Resolution (Å)			73.16–1.74
No. reflections			42,293 (2,308)
R_work/_ R_free_			0.174/0.209

**No. atoms**			

Protein			3,380
Ligand			52
Water			203

**B-FACTORS (Å2)**			

Protein			34.87
Ligand			38.07
Water			35.61

**RMSDs**			

Bond length (Å)			0.019
Bond angles (°)			1.899

**Ramachandran plot (%)**			

Favored region			98.0
Allowed region			2.0
Outlier region			0

Values in parentheses indicate highest-resolution shell.

a Anomalous.

**Figure 2:**
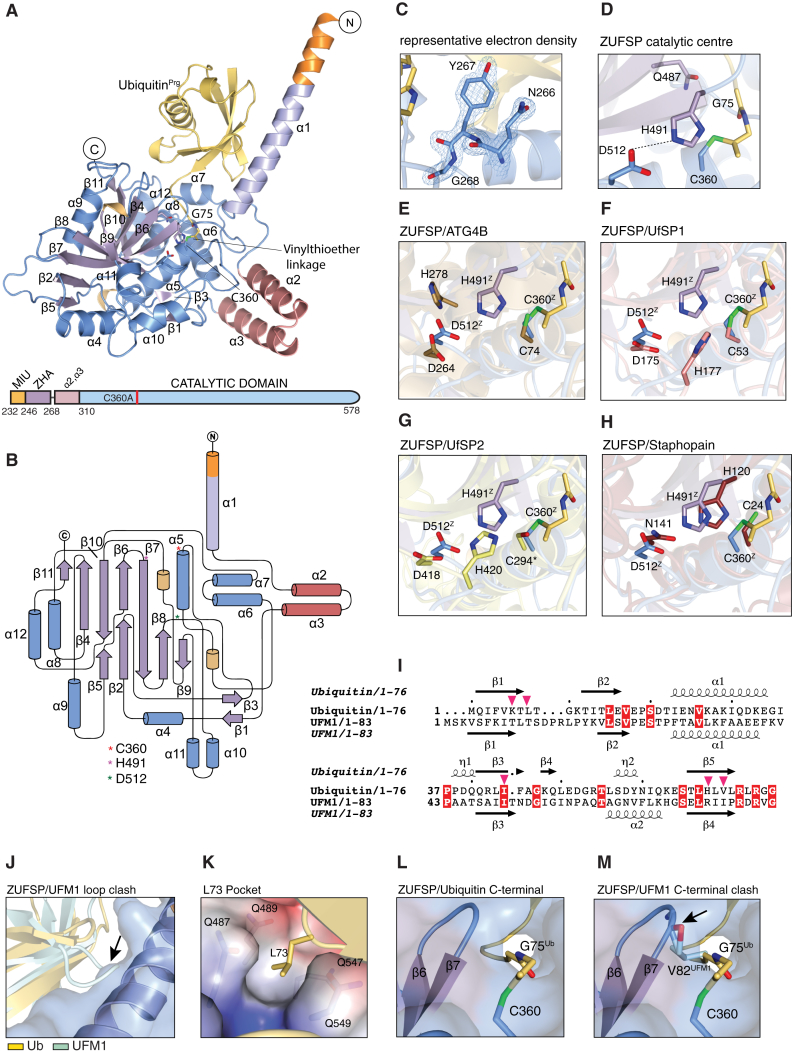
ZUFSP Is a Distinct Class of Deubiquitinating Enzymes (A) Overall structure of the ZUFSP catalytic core domain. The secondary structural elements, α helix, β strand, and 3–10 helix, are highlighted in actinium blue, light purple, and light orange, respectively, with covalently bound Ub (yellow). The vinylthioether linkage connecting Ub with the catalytic cysteine of ZUFSP is shown in stick format. The catalytic core domain is connected to the helical arm indicated as ZHA (light blue) via a helix-loop-helix motif (salmon red, α2 and α3). The N terminus highlighted in orange is part of the MIU motif. Below: schematic representation of the ZUFSP 232–578 construct. Colors depicting the domains correspond to colors of the structure. (B) Topology diagram showing ZUFSP architecture in 2D representation. The catalytic triad residues are indicated (asterisk). (C) Representative electron density. 2F_o_-F_c_ map of the residues corresponding to ZHA and the preceding loop contoured at 1σ. (D) Close-up view highlighting catalytic triad residues C360, H491, and D512 and the oxyanion hole forming residue Q487. (E–H) Close-up view of structure based alignments of ZUFSP with top matches based on the DALI server: Atg4b (PDB:2CY7) (E), UFSP1 (PDB: 2Z84) (F), UFSP2 (PDB: 3OQC) (G), and Staphopain (PDB:1CV8) (H). (I) Structural alignment of Ub with UFM1. The secondary structure elements for both Ub and UFM1 are shown. The Ub residues interacting with ZHA are indicated in asterisks. Fully conserved residues are shaded in red. (J) Steric clash of UFM1 β1-β2 loop with ZHA as seen from the structural alignment of UFM1 onto Ub (K) Electrostatic surface potential based representation shows the hydrophobic pocket formed by the aliphatic portions of Q487, Q489, Q547, and Q549. (L) The C terminus of Ub (yellow) in the narrow ZUFSP catalytic groove (M) Structural superposition of UFM1 (light blue). Bulky V82 of UFM1 clashes with the β6-β7 loop in ZUFSP. See also Figure S2.

The asymmetric unit contains one molecule of the complex, with clear discernible electron density for residues 236 to 578 of ZUFSP, and the entire Ub molecule (Figures 2A, 2C, S2A, and S2B). The catalytic domain of ZUFSP consists of three subdomains: a compact central subdomain that is flanked by two helical extensions perpendicular to each other (Figures 2A and 2B). Going from the core domain, α helices 2 and 3 (α2, α3) form the first extension. The second extension created by α1 forms a long helical arm, ZUFSP helical arm (ZHA) that protrudes away from the central domain. Intriguingly, only part of the MIU is ordered, and this does not mediate any interactions with Ub. Instead, the ZHA makes extensive contacts with the distal Ub, forming a unique UBD (see below).

Comparison with known structures in the Protein Data Bank (PDB) using the Dali server (Holm and Rosenström, 2010) did not identify any DUBs bearing similarity to ZUFSP. The closest matches are UFSP2 (PDB: 3OQC [Ha et al., 2011], Dali *Z* score 17.9, root-mean-square deviation [RMSD] 3.1 Å), UFSP1 (PDB: 2Z84 [Ha et al., 2008], Dali *Z* score 16.4, RMSD 2.9 Å), Atg4b (PDB: 2CY7 [Sugawara et al., 2005], Dali *Z* score 10.6, RMSD 3.3 Å), and Staphopain (PDB: 1CV8, Dali *Z* score 11.1, RMSD 3.1 Å) (Figures S2C–S2F). The catalytic triad in thiol proteases is made up of a catalytic Cys, a His, and usually an Asp/Asn residue that stabilizes the catalytic His (Clague et al., 2013). The crystal structure reveals that the catalytic triad in ZUFSP is made up of C360, H491, and D512 (Figure 2D). A conserved Gln Q487 forms the oxyanion hole that stabilizes the carbonyl oxygen atom of the scissile bond (Figure 2D). Overall, ZUFSP adopts a similar fold as UFSP1 and UFSP2 (Figures S2C and S2D). However, a close-up view reveals that the catalytic architecture of ZUFSP is distinct from that of Atg4b, UFSP1, and UFSP2 (Figures 2E–2G). Surprisingly, the catalytic center of ZUFSP closely resembles that of Staphopain, a CA clan cysteine protease from the bacterium *Staphylococcus aureus* (Figure 2H).

Hence, we classify ZUFSP to be a separate class of DUBs that forms a seventh family. Despite extensive sequence analyses to find other closely related members that could belong to the ZUFSP family, we failed to identify any, thus making ZUFSP the only member of this DUB class. ZUFSP is conserved in the eukaryotic domain and is present in various model organisms such as mouse (*Mus musculus*), frog (*Xenopus laevis*), zebrafish (*Danio rerio*), and the fission yeast *S. pombe* (mug105—meiotically upregulated gene 105) (Figure S3).

We next analyzed the structure to understand why ZUFSP does not react with UFM1 despite both Ub and UFM1 having a similar β-grasp fold (Figure 2I). Superposition of UFM1 onto Ub reveals that the β1-β2 loop of UFM1 sterically clashes with the ZHA (Figures 2J and S2H). In most DUBs, the flexible C terminus of Ub is stabilized by several interactions in the active site cleft of the DUB. Extensive hydrogen bonding and ionic interactions between R72 and R74 of Ub and D406, Q408, Q412, and E428 stabilize the C terminus of Ub. One important residue is L73 of Ub, which is accommodated in a hydrophobic pocket and is important for catalysis (Békés et al., 2013). In ZUFSP, this pocket is formed by the aliphatic portions of the side chains of Q487, Q489, Q547, and Q549 (Figures 2K and S2I). The equivalent residue of L73 in UFM1 is a negatively charged D80. Another significant difference between Ub and UFM1 lies in the C-terminal tail of the modifiers. The C-terminal residues of Ub, Gly75, and Gly76 are nestled within a narrow catalytic groove (Figure 2L). The equivalent residues in Ufm1 are Val82 and Gly83, and the bulky Val82 (Figure 2M) cannot be accommodated in this narrow groove, likely explaining why ZUFSP cannot be a UFM1 protease but is instead a DUB.

### ZHA Is a Distinct UBD

**Figure 3:**
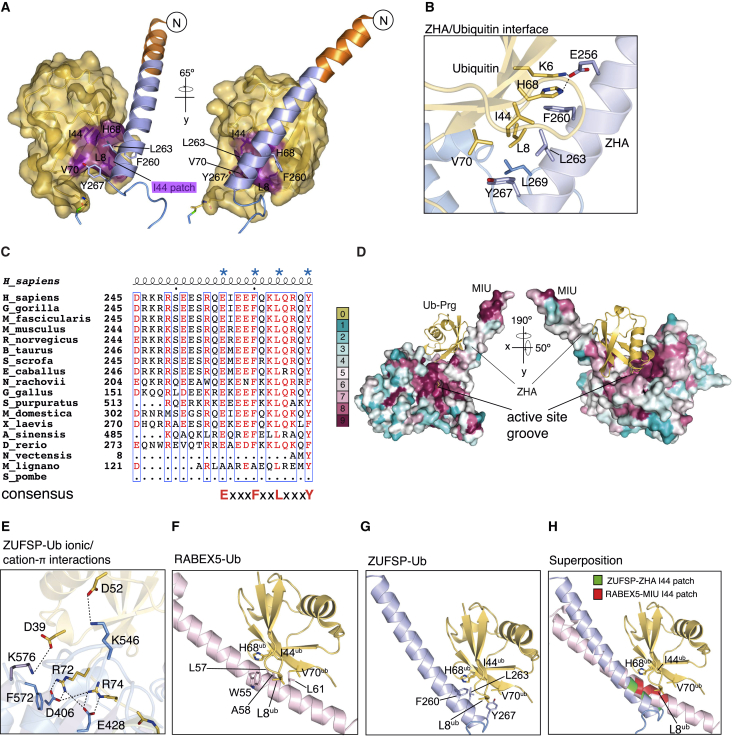
ZUFSP Helical Arm Is a UBD (A) ZHA-Ub interaction is predominantly hydrophobic. The interface shows the I44 patch (purple) on Ub (yellow) surface engaging with F260, L263, and Y267 residues of ZHA (cartoon representation, light blue). (B) The network of interactions between ZHA and Ub, which include hydrophobic interactions, a salt bridge between K6 and E256 of ZHA and cation-π pair (K6-F260) is depicted. (C) ZUFSP ZHA is conserved in evolution. Sequence alignment of ZHA from different organisms is shown. Secondary structure assignment is based on human ZUFSP. The ZHA residues interacting with Ub are highlighted with asterisks. Some species like *N. vectensis and S. pombe* altogether lack ZHA. (D) Surface representation showing conserved residues on the surface of ZUFSP based on the sequence alignment in Figure S3 generated with the Consurf server (http://consurf.tau.ac.il). The residues around the catalytic center and Ub interacting ZHA patch are conserved through evolution. (E) Interactions of distal Ub with ZUFSP. The Ub C-terminal residues R72 and R74 are involved in a network of ionic interactions with D406 and E428 of the catalytic domain. (F) Structure of the MIU of RABEX5 (pink) in complex with Ub (yellow) (PDB: 2FIF) shown in cartoon representation. (G) Structure of the ZHA of ZUFSP (blue) in complex with Ub (yellow) shown in cartoon representation. (H) Comparison of MIU and ZHA interactions with Ub. Superposition of structures shown in (F) and (G) aligned on Ub. See also Figure S3.

The crystal structure of ZUFSP in complex with Ub-Prg reveals several unique features about ZUFSP. The distal Ub interaction with the catalytic domain is extensive involving hydrophobic and polar interactions with a total buried surface area of 1,194 Å^2^. Strikingly, the conserved ZHA that extends out from the catalytic domain cradles the distal Ub by making extensive hydrophobic contacts with the Ile44 patch of Ub (Figure 3A). The main hydrophobic interactions are mediated by F260, L263, and Y267, which are all positioned on the same face of the ZHA, and these residues are conserved in evolution (Figures 3B–3D). In addition, K6 of Ub is involved in cation-π interactions with F260 and ionic interactions with E256 of the ZHA (Figure 3B). Lastly, L269, which is in the flexible α1-α2 loop, mediates hydrophobic interactions with L8 in the I44 patch of Ub (Figure 3B).

Further hydrogen bonding between R248, E252, Q259, and Q264 of ZHA and K63, I44, and A46 of Ub stabilize distal Ub interactions with the ZHA. Interestingly, while the Ub–ZHA interface mainly involves hydrophobic and ionic interactions, the interaction between Ub and the catalytic core is predominantly ionic in nature (Figure 3E). Many UBDs are helical domains that bind to Ub. Of these, the Ub interaction motif (UIM), MIU, and the double UIM (DUIM) are single helix domains (Hurley et al., 2006). We therefore compared the mode of interaction of Ub with ZHA, UIM, and MIU (Figures 3F–3H). Whereas the interactions of UIM and MIU with the I44 patch of Ub are centered around critical leucine and alanine residues, the key residues in ZHA are ExxxFxxLxxxY (Figures 3F and 3G). The mode of interaction of ZHA with Ub is reminiscent of that of MIU. However, compared to MIU, the Ub is rotated, and there is a clear difference in the way the I44 patch binds to the ZHA (Figure 3H). The interactions of ZHA with the I44 patch of Ub is mediated by residues distinct from an MIU, and the unique mode of interaction lead us to classify ZHA as a distinct single helix UBD.

### Identification of a UBZ Domain in ZUFSP

**Figure 4:**
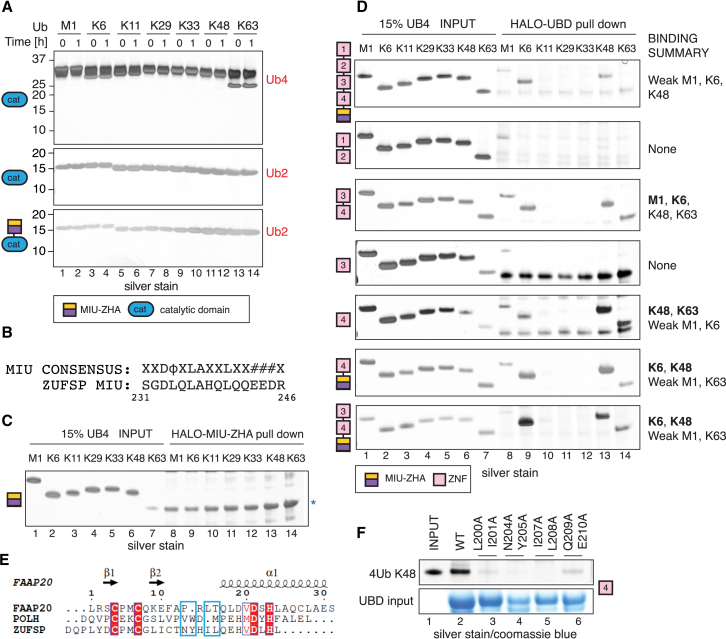
Dissecting PolyUb Binding of ZUFSP (A) DUB assays of ZUFSP^cat^ and ZUFSP^MIU-cat^ with the indicated polyUbs. Reaction products were separated by SDS-PAGE and silver stained. (B) Alignment of the MIU from ZUFSP with the consensus MIU motif (Penengo et al., 2006). (C) Ub pull-down assay where HALO-MIU-ZHA immobilized on HaloLink resin was incubated with tetra-Ub (UB4) of different linkages. Bound material was separated by SDS-PAGE and silver stained. Asterisks denote unspecific bands. (D) Indicated HALO-fusion proteins were incubated with UB4 and processed as in (B). Captured chain types are highlighted in the Binding Summary section. (E) ESPRIPT sequence alignment of ZNF4 from ZUFSP with UBZ of DNA polymerase η (POLH) and FAAP20. Residues highlighted within blue frames are important for ZUFSP ZNF4 polyUb binding. (F) Recombinant HALO-ZNF4 wild-type (WT) and various point mutants were analyzed for binding to tetra-K48 linked chains. Bound material was separated by SDS-PAGE and stained by Coomassie blue. See also Figure S4.

The crystal structure of ZUFSP reveals that its catalytic domain binds Ub. Therefore, we next sought to determine the types of polyUb it could cleave. We performed a DUB assay to compare cleavage of tetraUb of seven different linkage types. To our surprise, despite being reactive toward Ub-Prg, neither ZUFSP^Cat^ nor ZUFSP^MIU-Cat^ could cleave any of the polyUbs tested (Figures 4A and S4A). While the distal Ub binds to ZHA, an S1′ binding site for the proximal Ub is not obvious from the structure (Figure 2A). The MIU of ZUFSP, which has the conserved residues of a typical MIU motif is only partially ordered in the crystal structure and does not show any interaction with Ub, prompting us to question whether the MIU binds Ub (Figures 2A, 4B, and S2B). Certain MIUs, like that of MINDY1 (Kristariyanto et al., 2017), can bind to polyUb on their own. In contrast, most other individual MIUs do not bind polyUb and instead work together with other UBDs to enable binding (Husnjak and Dikic, 2012). When tested for polyUb binding, the MIU-ZHA fusion did not bind to any of the polyUbs tested (Figure 4C). Addition of MIU-ZHA to the catalytic domain does not confer activity to the DUB. Further, the positioning of the MIU makes it unlikely to be the S1′ site.

We therefore wondered whether the ZNFs in ZUFSP may bind Ub to provide the S1′ site and make ZUFSP an active DUB. The MIU-ZHA motifs together with the ZNFs (ZNF1–4) bind weakly to polyUb (Figure 4D), suggesting that one or more of the ZNFs bind Ub. To identify the ZNFs responsible for binding, we assayed polyUb binding of combinations or individual ZNFs (Figures 4D, S4B, S4C, S4D, and S4H). Our systematic analyses revealed that ZNF4 is the dominant polyUb binder in ZUFSP and the MIU may provide weak interactions that, together, form the polyUb binding module of ZUFSP (Figures S5H–S5J). These results using tetraUb pull-downs are further supported by pull-downs of ubiquitylated proteins from cell extracts (Figures S4F and S4G). Ub-binding ZNF (UBZ) is a type of Zn^2+^-coordinating β-β-α fold domain found in several proteins involved in DNA repair and transcriptional regulation (Husnjak and Dikic, 2012). Sequence analyses reveal that ZNF4 is a C2H2 type Zn^2+^ finger that is divergent from the well-characterized UBZ domains of POLH and FAAP20 (Toma et al., 2015) (Figure 4E), suggesting that ZNF4 of ZUFSP is an atypical UBZ. To further establish that it is a bona fide UBZ, we mutated residues in ZNF4, which we predicted based on sequence analyses to be involved in Ub recognition (Figure S4E). Indeed, these mutants completely disrupt polyUb binding, thus supporting the conclusion that ZNF4 is a UBD (Figure 4F).

### UBZ of ZUFSP Is Required for Catalytic Activity

**Figure 5:**
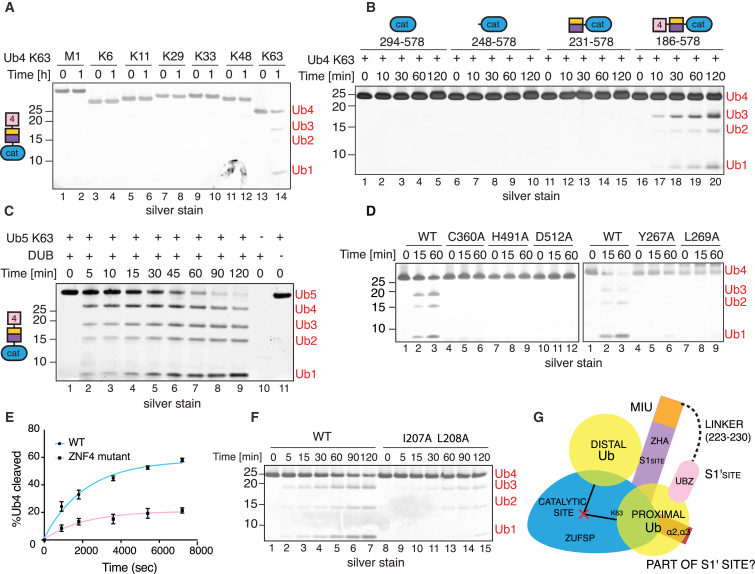
ZUFSP Catalytic Activity Requires the UBZ Domain (A) DUB assay of ZUFSP^ZNF4-MIU-cat^. Purified protein was incubated with each Ub4 chain type for the indicated time points, and reaction products were separated by SDS-PAGE and silver stained. (B) DUB assay as in (A) comparing DUB activity of the indicated ZUFSP constructs. (C) DUB assay monitoring K63-pentaUb cleavage by ZUFSP^ZNF4-MIU-Cat^, with reaction products visualized as in (A). (D) DUB assay comparing activity of the indicated ZUFSP mutants at cleaving K63-tetraUb (E) Comparison of DUB activities of ZUFSPZNF4-MIU-Cat with UBZ mutant that cannot bind ubiquitin. Percentage of cleaved tetraUb was quantified from Sypro Ruby-stained gels. Data from three independent experiments were fitted using nonlinear regression, one phase exponential decay. SD error bars are shown. (F) DUB assay as in (D) monitoring cleavage of K63-linked tetraUb. (G) Model depicting substrate binding and catalysis in ZUFSP. The distal Ub is stabilized by contacts with the ZHA domain (violet) and the catalytic core (blue). The polyUb binding UBZ domain (ZNF4) (pink) is required for catalytic activity and we suggest that it may form the proximal Ub binding S1’ site. See also Figure S5.

Having identified a UBZ domain in ZUFSP that binds polyUb, we asked whether the addition of this domain would confer DUB activity to the catalytic domain of ZUFSP. Compared to the minimal catalytic domain, which does not cleave polyUb, addition of the UBZ domain converted ZUFSP into an active DUB with remarkable specificity for cleaving K63-linked polyUb (Figures 5A and 5B). A detailed time course reveals that polyUbs of different lengths are produced upon cleavage of long K63-linked polyUbs, suggesting that ZUFSP is an endo-DUB (Figures 5C, S5B, and S5C). It is conceivable that the UBZ may form the proximal Ub binding site thus explaining DUB activity of ZUFSP only in the presence of the UBZ. If this idea is correct, then we predict that changing the location of the UBZ would hinder its positioning and thereby its role as the S1′ site. Indeed, changing the position of UBZ by fusing it to the C terminus of ZUFSP resulted in loss of DUB activity, supporting our notion that the UBZ may form the proximal Ub binding site (Figure S5A). Importantly, mutating residues in the UBZ that disrupt Ub binding (Figure 4F) lead to impaired DUB activity (Figures 5E and 5F), thus highlighting the importance of the UBZ domain for the enzyme activity of ZUFSP.

Furthermore, our finding that the UBZ is required for DUB activity allowed us to test mutants to validate our observations based on the crystal structure using DUB assays. Mutation of C360, H491, D512, or Q487 to Ala completely abolished catalytic activity, supporting the insights from the crystal structure that C360, H491, and D512 are the main catalytic residues and Q487 forms the oxyanion hole (Figures 5D and S5D). Mutating Y267 and L269 to Ala also abolished DUB activity, highlighting the importance of the ZHA and distal Ub binding for catalysis (Figure 5D). In summary, the UBDs in ZUFSP we have here identified, the ZHA and UBZ, form the distal and the putative proximal Ub binding sites, respectively, to together enable the DUB to selectively cleave K63-linked polyUb (Figure 5G).

### ZUFSP Is a Putative Regulator of DNA Replication and Repair

**Figure 6:**
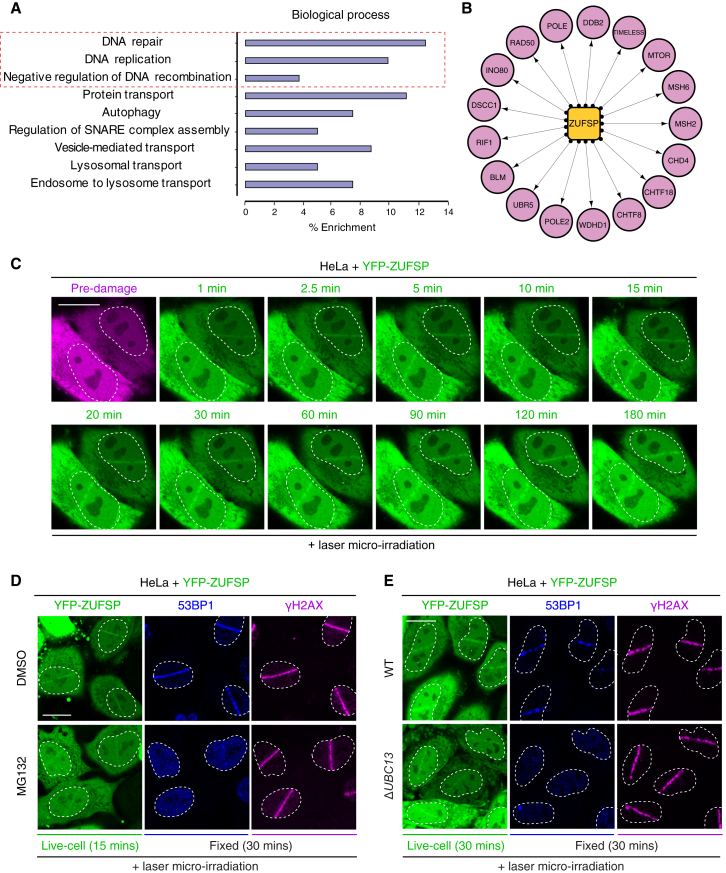
ZUFSP Is a Putative DNA Replication and Repair Factor (A) Immunoprecipitation of endogenous ZUFSP reveals distinct biological functions. DAVID analysis for Gene Ontology Biological Processes (GOBP) showing enrichment for the top hits. (B) Subset of ZUFSP interaction map showing proteins involved in DNA repair, DNA replication, and negative regulation of DNA recombination. (C) HeLa cells were transfected with YFP-ZUFSP, subjected to laser micro-irradiation, and imaged at the indicated time. Scale bar, 10 μm. (D) HeLa cells were transfected with YFP-ZUFSP, pre-treated with DMSO or MG132 (3 hr), subjected to laser micro-irradiation, and imaged at the indicated time and then fixed and processed for immunostaining with the indicated antibodies. Scale bar, 10 μm. (E) HeLa wild-type (WT) of *ΔUBC13* cells were transfected with YFP-ZUFSP, subjected to laser micro-irradiation, and imaged at the indicated time and then fixed and processed for immunostaining with the indicated antibodies. Scale bar, 10 μm. See also Figure S6.

To gain insights into the biological roles of this newly identified K63-selective DUB, we raised antibodies against human ZUFSP. As a first step, we analyzed the subcellular localization of ZUFSP by biochemical fractionation, which revealed that ZUFSP is mostly present in the nuclear fraction, suggesting a function in the nucleus (data not shown). Next, we immunoprecipitated endogenous ZUFSP from the nuclear fractions of HEK293 cells and performed mass spectrometry analysis to identify interacting proteins. Interestingly, pathway analysis revealed that several of the identified ZUFSP interactors are proteins that regulate DNA replication and repair (Figures 6A, 6B, S6E, and S6F). To interrogate the involvement of ZUFSP in the latter, we first monitored whether it localizes to sites of DNA damage, as this is a hallmark of proteins involved in DNA repair. Interestingly, ZUFSP is rapidly recruited to DNA lesions following laser micro-irradiation (within 1 min) and also persists for more than 3 hr (Figure 6C). Next, to determine whether Ub signaling impacts ZUFSP recruitment, cells were pre-treated with the proteasome inhibitor MG132, which reduces free Ub pools and impairs Ub signaling at DNA lesions (Dantuma et al., 2006). Proteasome inhibition leads to a marked inhibition of total and K63-linked polyUb (Figures S6A and S6C), which correlated with a marked reduction of YFP-ZUFSP accrual at DNA lesions, suggesting a largely Ub-dependent component of ZUFSP recruitment (Figure 6D). Given our above finding that ZUFSP is a K63-specific DUB, we genetically ablated *UBC13*, the Ub-conjugating (E2) enzyme that generates majority of the K63-linked polyUb in response to DNA damage (Figure S6D). We found that loss of K63-linked polyUb impairs optimal recruitment of YFP-ZUFSP to DNA lesions but is not the only determinant for its localization to damage sites, in line with our *in vitro* findings that ZUFSP can bind multiple polyUb types (Figures 6E, 4D, S6B, S5H, and S5I). Collectively, these findings suggest that ZUFSP is a putative DNA repair and/or replication factor involved in Ub signaling at DNA lesions.

### ZUFSP Is Required for Genome Stability

**Figure 7:**
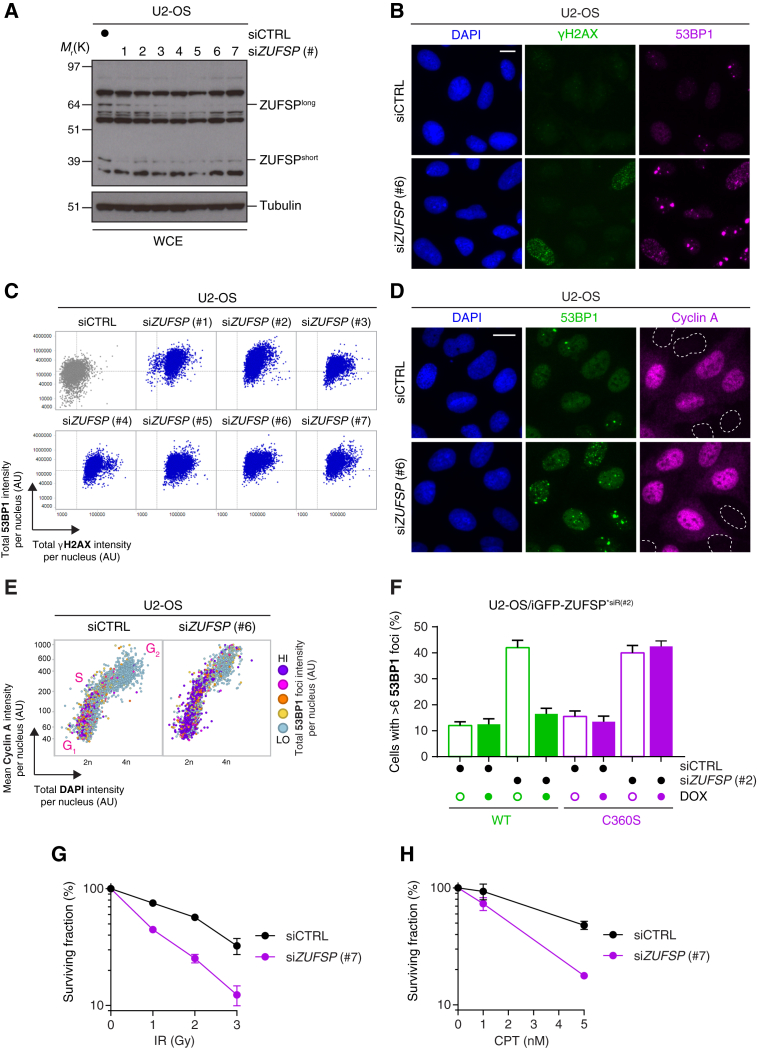
ZUFSP Prevents Spontaneous DNA Damage and Promotes Cellular Survival in Response Exogenous DNA Damage (A) U2-OS cells were transfected with control (siCTRL) or ZUFSP (siZUFSP) siRNAs for 72 hr and then lysed and analyzed by immunoblotting with the indicated antibodies. (B) U2-OS cells were transfected with the indicated siRNAs for 72 hr and then subjected to pre-extraction and fixation and processed for immunostaining with the indicated antibodies. Scale bar, 10 μm. (C) QIBC analysis of chromatin-bound γH2AX and 53BP1 from (B). A representative experiment is shown from at least n = 3 independent biological experiments. (D) U2-OS cells were transfected with the indicated siRNAs for 72 hr and then fixed and processed for immunostaining with the indicated antibodies. Scale bar, 10 μm. (E) QIBC analysis from the samples shown in (D) using the combination of cyclin A and DAPI signals to differentiate cell-cycle phases, with coloring indicating the total 53BP1 foci intensity per nucleus. (F) U2OS cells were treated with siCTRL or si*ZUFSP* (#2) siRNAs and with or without doxycycline (DOX) to induce expression of siRNA-resistant (^∗siR#2^) GFP-ZUFSP wild-type (WT) or C360S alleles. Cells were processed for immunostaining and enumerated for 53BP1 positivity. Data represent mean ± SEM from two biologically independent experiments using technical duplicates per data point. (G) Clonogenic survival of U2-OS cells transfected with the indicated siRNA and then treated with various doses of ionizing radiation (IR). Data represent mean ± SEM from two biologically independent experiments using technical triplicates per data point. (H) As for (F), but with camptothecin (CPT). See also Figure S7.

To explore a functional role for endogenous ZUFSP in DNA repair, we depleted ZUFSP in human cancer cells using a panel of small interfering RNAs (siRNAs) (Figure 7A). We consistently observed that cells depleted of ZUFSP exhibit increased chromatin loading of the DNA damage protein 53BP1 together with increased γH2AX signaling, suggesting that ZUFSP depleted cells undergo spontaneous DNA damage (Figure 7B). Quantitative image-based cytometry (QIBC) (Toledo et al., 2013) analysis of total γH2AX and 53BP1 signal in the chromatin fraction revealed a consistent DNA damage phenotype upon ZUFSP depletion using the entire panel of siRNAs (Figures 7C and S7A). A previous RPA proteomic screen identified ZUFSP in RPA co-immunoprecipitates (Tkáč et al., 2016), which we also confirmed (Figure S7B). However, ZUFSP recruitment to DNA lesions was independent of prior RPA loading, as depletion of CtIP did not impact YFP-ZUFSP recruitment to DNA lesions (Figure S7C). Based on our identification of DNA replication and repair factors in the ZUFSP interactome (Figure 6A) and the interaction with RPA, we reasoned that the DNA damage phenotype might have arisen from problems encountered during DNA replication. Using cyclin A as a marker for S/G_2_-phase and 53BP1 as a DNA damage marker, we observed that both G_1_- and S/G_2_-phase cells displays increased 53BP1 signal in ZUFSP depleted cells, indicative of increased DNA breaks in both these cell-cycle phases (Figure 7D). To extend these findings further, we used QIBC analysis to determine how the 53BP1 signal varied across the cell cycle from thousands of single cells (Figure 7E). Quantification of total 53BP1 signal, as measured from the sum of 53BP1 foci intensity per nucleus, revealed the highest 53BP1 levels in G_1_- and S-phases, which suggest that DNA damage suffered during ongoing DNA replication is inefficiently repaired in ZUFSP depleted cells and transmitted to the next cell cycle (Figure 7E). Next, we engineered cells lines to inducibly express either wild-type (WT) or catalytically inactive (C360S) siRNA-resistant ZUFSP alleles (Figures S7D, S5D, and S7E). While WT ZUFSP effectively rescued the DNA damage phenotype in ZUFSP depleted cells, the C360S mutant was unable to, suggesting that the DUB activity of ZUFSP is required to prevent spontaneous DNA damage in cells (Figure 7F). Last, we analyzed the resilience of ZUFSP depleted cells to exogenous DNA damage reagents. Relative to controls, ZUFSP depleted cells displayed increased sensitivity to both ionizing radiation (IR) and the chemotherapeutic drug camptothecin (CPT; topoisomerase I inhibitor) (Figures 7G and 7H), underlining a broader role for ZUFSP in DNA repair. Collectively, our data suggest that ZUFSP is a distinct DUB class that plays an important role in maintaining genome stability both during normal ongoing DNA replication and in response to exogenous DNA damage.

## Discussion

Here, we identify ZUFSP as a Ub-specific DUB that selectively cleaves K63-linked polyUb. This discovery was possible due to the application of activity-based DUB probes. Such probes have been instrumental in the identification of DUBs and in investigating Ub signaling (Borodovsky et al., 2002, Jahan et al., 2016). However, the recent development of better probes (Ekkebus et al., 2013), and our development of a more robust approach, led to the identification of ZUFSP as a putative DUB. Further, we identify distinct subsets of DUBs in different cell lines. Such powerful unbiased analyses of DUB activity in different tissues and primary cells has the potential to provide insights into regulation of Ub signaling in specific cell types and in response to different cellular stimuli. Our identification of another family of DUBs highlights that there is much more to be learned about the Ub system. Notably, the use of DUB probes is presently limited to thiol proteases and the development of probes that work with metalloproteases will significantly advance our understanding of regulation of metallo-DUB activity and may also reveal hitherto unknown Ub-specific metalloproteases.

Our results point to important roles for ZUFSP in the cellular response to replication stress. Hence, it is conceivable that the activity of ZUFSP has to be regulated. Being a modular protein, we suggest that there are several layers of regulation controlling the activity of ZUFSP. We have here identified two unannotated UBDs within ZUFSP, the helical ZHA juxtaposed to the catalytic domain that binds the distal Ub and the UBZ domain that binds polyUb. These findings increase the number of known UBDs and the ways by which Ub signals are decoded. We propose that the UBZ forms the S1′ site to make ZUFSP a functional DUB. Intriguingly, the UBZ is an atypical one that binds polyUb and so it is likely that it has two Ub binding sites located within the same domain, reminiscent of Npl4 ZNF (NZF) domains (Kristariyanto et al., 2015a). The UBZ domain thus joins a growing list of small domains that are capable of binding polyUb on their own (Husnjak and Dikic, 2012).

Surprisingly, the annotated UBD, i.e., the MIU motif, despite having all the canonical residues of a typical MIU motif, does not bind strongly to Ub. One possibility is that this MIU is a weak Ub binder that on its own does not bind Ub. The positioning of the MIU adjacent to ZHA suggests that it may serve as the S2 binding site. Interestingly, modeling a complete MIU-Ub interaction in ZUFSP positions the Ub in such a way that the C terminus of the Ub bound to MIU points toward K63 of the ZHA-bound Ub. Distance restraints suggest that only a K63-linked diUb can bind to ZHA-MIU, making it further likely that the MIU forms the S2 binding site (Figure S7F). Indeed, mutation of the MIU to disrupt Ub binding impacts on the efficiency of ZUFSP to cleave polyUb (Figures S5E and S5F). DUBs depend to varying extents on the different Ub binding sites they have (Mevissen and Komander, 2017). For instance, the S2 site in the DUB SARS PLpro is a dominant Ub binder and dictates specificity and activity of the DUB (Békés et al., 2016). In contrast, our data show that ZUFSP relies more on S1 and S1′ Ub recognition, with a minor contribution from the proposed S2 binding site. Having unequivocally demonstrated that ZUFSP is a DUB and not a UFM1 peptidase, we propose renaming ZUFSP to ZUP1 (Zinc finger containing Ub Peptidase 1).

Proteomic approaches have identified thousands of proteins that are ubiquitylated in response to DNA damage, although we are only just beginning to discover whether and how these signaling events are functionally important *in vivo*. (Elia et al., 2015, Povlsen et al., 2012). Promisingly, the Ub system has become a prominent target for drug discovery to treat cancers, with DUB inhibitors now progressing into clinical trials (Harrigan et al., 2018, Huang and Dixit, 2016, Pinto-Fernandez and Kessler, 2016). However, our understanding of Ub signaling regulation by DUBs within a cellular context remains under investigated. Our findings suggest that ZUFSP has a major role at stalled replication forks. The ZUFSP interactome identified several important components of the replisome, as well as known DNA repair factors found at stalled replication forks. Furthermore, loss of ZUFSP in human cancer cells led to increased endogenous DNA damage in these cells, and we could further show that this endogenous DNA damage originates in S-phase. Third, ZUFSP is required for cellular resilience to DNA damage with replication-dependent components. Collectively, we propose that ZUFSP is required for cellular responses to DNA replication stress.

Going forward, defining mechanistically how the biochemical activity of ZUFSP is linked to the replication stress phenotype is of paramount importance to understand. With its exquisite selectivity for cleaving K63-linked polyUb, it is plausible that ZUFSP regulates K63 ubiquitylation following replication stress. Alternatively, ZUFSP may be recruited via its UBZ domain to K6, K48, or K63 chains formed at sites of damage to subsequently cleave K63-chains from substrates. Being key for the activity of ZUFSP, it is tempting to speculate that the UBZ domain mediates substrate recruitment via binding to polyUb and as the S1′ site enabling cleavage of K63-linked chains. While we have identified a network of ZUFSP-associated proteins implicated in DNA replication and repair, it will be essential to determine which, if any, of these interactors are bona fide substrates of ZUFSP. However, identifying substrates of DUBs is not trivial and in fact cellular substrates for a vast majority of DUBs are unknown (Leznicki and Kulathu, 2017). Studying how dysregulated Ub signaling of ZUFSP substrates causes replication stress is likely to reveal fresh insights into the role of ubiquitylation in the cellular responses to DNA replication stress. Given that loss of ZUFSP in human cancer cells both causes endogenous DNA replication stress and sensitizes cells to further exogenous DNA damage, it will now be important to understand whether, and how, ZUFSP loss synergizes with defects in other DDR pathways.

## STAR★Methods

### Key Resources Table

**Table:** 

REAGENT or RESOURCE	SOURCE	IDENTIFIER
**Antibodies**		

Mouse anti-53BP1	Millipore	Cat# MAB3802, RRID:AB_11212586
Mouse anti-FLAG HRP	Sigma	Cat# A8592, RRID:AB_439702
Mouse anti-γH2AX (Ser139)	Biolegend	Cat# 613402, RRID:AB_315795
Mouse anti-Ub (FK2)	Enzo	Cat# BML-PW8810, RRID:AB_10541840
Rabbit anti-53BP1	Santa Cruz Biotechnology	Cat# sc-22760, RRID:AB_2256326
Rabbit anti-Cyclin A	Santa Cruz Biotechnology	Cat# sc-751, RRID:AB_631329
Rabbit anti-GFP	Abcam	Cat# ab290, RRID:AB_303395
Rabbit anti-γH2AX (Ser139)	Abcam	Cat# ab81299, RRID:AB_1640564
Rabbit anti-RFWD3	Bethyl	Cat# A301-397A, RRID:AB_961090
Rabbit anti-SMARCAL1	Bethyl	Cat# A301-616A, RRID:AB_1211349
Rabbit anti-Tubulin	Abcam	Cat# ab184970
Rabbit anti-UBC13	Cell Signaling Technology	Cat# 4919S, RRID:AB_2211168
Rabbit anti-Ub (K63-specific, Apu3)	Millipore	Cat# 05-1308, RRID:AB_1587580
Rabbit anti-Ub	Dako	Cat# Z0458, RRID:AB_2315524
Rabbit anti-UFM1	Abcam	Cat# ab109305, RRID:AB_10864675
Rabbit anti-ZUFSP	Sigma	Cat# HPA044426, RRID:AB_10960887
Rabbit anti-ZUFSP	Custom (Eurogentec)	N/A

**Bacterial and Virus Strains**		

pLenti CMV/TO Hygro DEST	Addgene	Cat# 17291
pLenti CMV TetR Blast	Addgene	Cat# 17492
pENTR4-GFP-C1	Addgene	Cat# 17396
pDONR221	Invitrogen	Cat# 12536017
pDEST12.2	Invitrogen	Cat# 11808011
pYFP DEST	In house	N/A
pFLAG-CMV DEST	In house	N/A

**Chemicals, Peptides, and Recombinant Proteins**		

MG132	Merck Millipore	Cat# 474790
Propargylamine	Sigma Aldrich	Cat# P50900-5G
Selenomethionine	Molecular Dimensions	Cat# MD12-503B
SureBeads Protein A Magnetic Beads	Bio-Rad	3161-4011
HaloLink Resin	Promega	G1915

**Deposited Data**		

Mass spectrometry proteomics data	ProteomeXchange Consortium via PRIDE repository	http://proteomecentral.proteomexchange.org
ZUFSP Structure factor and coordinates files	RCSB-PDB	https://www.rcsb.org/
Microscopy images	Mendeley	https://data.mendeley.com/datasets/hs6fgdvdg3/draft?a=8e80899a-da80-4a34-a0c9-3d045f523f61
Gel images	Mendeley	https://data.mendeley.com/datasets/x7p9vk5cvt/draft?a=d198a3ad-9673-453c-b036-6045c75380e9

**Experimental Models: Cell Lines**		

U2 O-S	ATCC	Cat# HTB-96, RRID:CVCL_0042
HeLa S3	ATCC	Cat# CCL-2.2, RRID:CVCL_0058
293	ATCC	Cat# CRL-1573, RRID:CVCL_0045
Jurkat	ATCC	Cat# TIB-152, RRID:CVCL_0367

**Oligonucleotides**		

pDONR221-ZUFSP FWD: GGGGACAAGTTTGTAC AAAAAAGCAGGCTTCATGCTTTCCTGTAATATTTGTGG	This paper	N/A
pDONR221-ZUFSP REV: GGGGACCACTTTGTACAAGA AAGCTGGGTATCAAGGAATCTTCTCGGCTGT	This paper	N/A
ZUFSP siRNA-resistant (#2) FWD (i): CATATGTACAAATT ATCACATACTTCAGGAACATGTT	This paper	N/A
ZUFSP siRNA-resistant (#2) REV (i): AACATGTTCCTGAAG TATGTGATAATTTGTACATATG	This paper	N/A
ZUFSP siRNA-resistant (#2) FWD (ii): ACAAATTATCACATA TTGCAAGAACATGTTGACTTG	This paper	N/A
ZUFSP siRNA-resistant (#2) REV (ii): CAAGTCAACATGTTC TTGCAATATGTGATAATTTGT	This paper	N/A
pX459-UBC13 FWD: CACCGGGCGTTGCTCTCATCTGGTT	This paper	N/A
pX459-UBC13 REV: AAACGATGAGAGCAACGCCCGTTAC	This paper	N/A
siCTRL: (siGENOME Non-targeting siRNA pool #1)	Dharmacon	Cat# D-001206-13
siCTRL: GGGAUACCUAGACGUUCUA		N/A
si*ZUFSP* (#1): AUAUGGAACUUCAGAUAAC	This study	N/A
si*ZUFSP* (#2): UUACCAUAUUCUUCAGGAA	This study	N/A
si*ZUFSP* (#3): GGUCACAGUCGAACUGUUA	This study	N/A
si*ZUFSP* (#4): CAGUCGAACUGUUAUUGGA	Ambion	Cat# s48009
si*ZUFSP* (#5): GGAAGACUGUGAUCAACCA	Ambion	Cat# s48010
si*ZUFSP* (#6): GGAACUUCAGAUAACAAGA	Ambion	Cat# s48011
si*ZUFSP* (#7, siGENOME SMARTpool):	Dharmacon	Cat# M-015894-00

**Software and Algorithms**		

Spotfire	Tibco	https://spotfire.tibco.com RRID:SCR_008858
CellProfiler	Broad Institute	http://cellprofiler.org RRID:SCR_007358
MaxQuant v 1.6.0.13	Tyanova, S. et al., 2016	http://www.coxdocs.org/doku.php?id=maxquant:common:download_and_installation#downloadRRID:SCR_014485
Saint Express 3.6.1	Teo et al., 2014	https://sourceforge.net/projects/saint-apms/files/
Prism	Graphpad	https://www.graphpad.com/scientific-software/prism/ RRID:SCR_002798
XDS	Kabsch, 2010	http://xds.mpimf-heidelberg.mpg.de/
CCP4 interface version 7.0.048	Winn et al., 2011	http://www.ccp4.ac.uk/
AIMLESS	Evans and Murshudov, 2013	http://www.ccp4.ac.uk/html/aimless.html RRID:SCR_015747
MAD phasing mode of Auto-Rickshaw	Panjikar et al., 2005	http://www.embl-hamburg.de/Auto-Rickshaw/
COOT	Emsley et al., 2010	http://www2.mrc-lmb.cam.ac.uk/personal/pemsley/coot/ RRID:SCR_014222
REFMAC5	Murshudov et al., 1997	http://www.ccp4.ac.uk/html/refmac5/description.html RRID:SCR_014225
PDB-REDO	Joosten et al., 2014	http://www.cmbi.ru.nl/pdb_redo/
PyMOL		https://pymol.org/2/ RRID:SCR_000305

### Contact for Reagent and Resource Sharing

Requests for further information or reagents should be directed to the Lead Contact and corresponding author, Yogesh Kulathu (ykulathu@dundee.ac.uk).

### Method Details

#### Molecular Biology

Most of the cDNA constructs used in this study were generated by the Cloning team of Division of Signal Transduction Therapy (DSTT), MRC Protein Phosphorylation and Ubiquitylation Unit, University of Dundee, United Kingdom (Table S1). In addition, full length human ZUFSP cDNA was cloned into the pDONR221 vector (Thermo Fisher), and subsequently used to generate N-terminal YFP-ZUFSP using the LR Clonase II enzyme mix (Thermo Fisher). Point mutants were produced in pDONR221-ZUFSP using Phusion High-Fidelity DNA Polymerase (New England Biolabs). Full-length RPA1, RPA2 and RPA3 cDNAs were cloned into pDONR221 before generating FLAG-RPA1, FLAG-RPA2 and FLAG-RPA3 or untagged-RPA1, RPA2 and RPA3.

#### Protein expression and Purification

##### GST Purification

*E.coli* BL21 cells expressing recombinant GST-fusion proteins were grown at 37°C in 2xTY medium and expression was induced at OD 0.6 with 300 μM IPTG, followed by overnight growth at 18°C. Medium was supplemented with 200 μM ZnCl_2_ for expression of ZNF-containing proteins. Cells were lysed in GST Lysis Buffer (50 mM Tris pH 7.5, 300 mM NaCl, 10% glycerol, 0.075% β-mercaptoethanol, 1 mM benzamidine, 1 mM AEBSF and protease inhibitor cocktail (Roche)). Lysate was sonicated and centrifuged for 30 min at 30,000 x g at 4°C. Subsequently, the lysate was incubated with GSH beads (DSTT) for 2 h at 4°C. The resin was washed extensively with high salt buffer (25 mM Tris pH 7.5, 500 mM NaCl, and 1 mM DTT), followed by low salt buffer (25 mM Tris pH 7.5, 150 mM NaCl, 10% glycerol, and 1 mM DTT). Bound protein was eluted by overnight C3 protease cleavage in low salt buffer at 4°C. Protein was concentrated using a centrifugal filter (Amicon) and either further purified by size exclusion chromatography or frozen in liquid nitrogen and stored at −80°C.

#### Chitin binding domain purification

Chitin binding domain followed by intein fusions with Ub/UBL of interest was expressed in E.coli BL21 and expression was induced with 300 μM IPTG at OD 0.4-0.6, followed by overnight growth at 18°C. Cells were lysed in CBD lysis buffer (20 mM Na_2_HPO_4_ pH 7.2,

200 mM NaCl, 0.1 mM EDTA) on ice for 10 min, sonicated and then centrifuged for 30 min at 30,000 x g at 4°C. Subsequently, the clarified lysate was incubated with 40 mL of chitin resin for 4 h at 4°C. Next, resin was washed with 800 mL of CBD lysis buffer, then 100 mL of the elution buffer (20 mM Na_2_HPO_4_ pH 6, 200 mM NaCl, 0.1 mM EDTA) (minus MESNA, until the pH was reduced to 6). Then, 50 mL of elution buffer (with MESNA) was added to the resin. The lysate was incubated with the resin overnight on a roller at 4°C. The eluate was harvested and a second elution performed for 6 hours in a 20 mL volume. Pooled eluate was concentrated to approximately 6 mL and either directly used for reaction with alkylating agent or frozen and kept at −80°C.

#### Purification of ZUFSPCat-Ub-Prg complex for crystallization

Ub reactive probe with propargylamine as a warhead was purified as described elsewhere (Abdul Rehman et al., 2016). The ZUFSP-MIU catalytic domain construct spanning 231-578 was used for protein expression. For heavy atom labeling, M9 minimal media supplemented with glucose, vitamins and amino acids with the exception of L-methionine was prepared. In the supplemented M9 medium, L-Selenomethionine was added at the final concentration of 30 mg/l. The overnight pre-culture was grown in LB medium at 37°C and harvested at 2500 rpm for 5-8 minutes followed by resuspension in M9 supplemented media. Following inoculation, bacterial cultures were induced 0.7 OD_600_ with 300 μM IPTG. Post induction, the temperature was lowered to 18°C and cells were left shaking for another 20 hours before harvest. Protein was purified according to GST-purification step as described. The cleaved protein was eluted in a buffer containing 25 mM HEPES pH 7.5, 150 mM NaCl, 10% glycerol, and 1 mM DTT. Further, the protein was mixed with 3 fold excess of propargylated Ub and incubated for overnight on a roller at 4°C. Following cation exchange chromatography to segregate ZUFSP^Cat^-Ub^Prg^ complex from the unreacted ZUFSP^Cat^ domain, gel filtration chromatography on Superdex G75 was done as the last step in the purification. All the fractions post SDS-PAGE run were pooled and concentrated for crystallization trials.

#### Crystallization, Data Collection and Structure Determination

The ZUFSP MIU-catalytic domain construct (residues 231-578) was expressed in *E.coli* BL21 cells. Proteins were purified as described in STAR Methods. Recombinant ZUFSP^MIU-cat^ protein was eluted in 25 mM HEPES pH 7.5, 150 mM NaCl, 10% glycerol, and 1 mM DTT pH 8 and then mixed in a 1: 3 ratio with propargylated Ub (Ub-Prg) and incubated for 12 h at 4°C. ZUFSP^MIU-cat^-Ub-Prg complexes were purified and crystallized. Initial ZUFSP-Ub-Prg (native) crystals were obtained using sitting-drop vapor diffusion method against the well solution containing 4% v/v Tacsimate pH 5.0 and 12% w/v Polyethylene glycol 3,350 (14 mg/ml). The crystals were flash frozen in cryoprotectant containing 4% v/v Tacsimate pH 5.0, 30% glycerol and 5% ethylene glycol. The crystals diffracted X-rays to 1.74Å at I04 beamline, DLS, UK. After unsuccessful attempts of using the Ub as a search model to solve the structure by molecular replacement, the ZUFSP-Ub-Prg was Selenomethionine labeled for anomalous phasing. The crystals were grown in sitting drop 24 well plates against the well solution of 4% v/v Tacsimate pH 5.0 and 12% w/v Polyethylene glycol 3,350 (8.5 mg/ml). The crystals were flash frozen in the cryoprotectant as used above for the native crystals. The MAD datasets for two different wavelengths 0.97264 (peak) and 0.97923 (inflection) were collected from different part of the same crystal at ID29 beamline, ESRF, France. The anomalous data were processed using XDS (Kabsch, 2010) and then scaled using AIMLESS (Evans and Murshudov, 2013). The structure was solved using the two wavelength MAD phasing mode of Auto-Rickshaw: the EMBL-Hamburg automated crystal structure determination platform (Panjikar et al., 2005). The partially built model obtained was further submitted to the MRSAD pipeline. The complete model was obtained after iterative building and refinement with COOT (Emsley et al., 2010) and REFAMC5 (Murshudov et al., 1997) (Winn et al., 2011). The anomalous and native datasets were sufficiently isomorphous to allow refinement of the model against the native dataset. The final structure was re-refined using PDB-REDO (Joosten, R.P. et al., 2014). The final data collection and refinement statistics for the ZUFSP-Ub-Prg complex structure is shown in Table 1. All the figures were made using PyMOL (http://pymol.org).

#### Sequence and Structural analyses:

Multiple sequence alignments were created in Jalview (Waterhouse et al., 2009) by using the MAFFT package in a default mode (Katoh and Toh, 2010). All the structure based alignments were done in Coot using SSM (secondary structure matching) or LSQ (least square comparison) methods. The electrostatic surface charge distribution of ZUFSP was calculated using ABPS plugin in PyMOL.

#### Generation of activity-based probes (ABPs)

Probes were synthesized based on intein-based chemical ligation (Borodovsky et al., 2002) and purified on Chitin Resin (BioLabs). Eluates were adjusted to pH 8.0 with 0.5M NaOH followed by addition of 250 mM propargylamine and incubated for 6 h at 18°C in the dark. Propargylated Ub was separated from unreacted propargylamine and other impurities by gel filtration chromatography and suspended in PBS, concentrated and stored at −80°C.

#### DUB profiling

The quality of the ABP was tested in a small-scale reaction by mixing specified DUBs with the propargylated probes in a 1:3 molar ratio in 10 μL reactions and incubated for specified time at 37°C. 3 μL of 4X LDS was added to stop the reaction. Samples were separated on 4%–12% gradient gels (Thermo Fisher) and visualized with Coomassie Blue stain.

For profiling DUBs from cells, 10 μM probe was coupled to 100 μL of HaloLink-resin as bait. 10 mg of cell lysate was pre-cleared with HaloLink resin and DUB capture was performed using HaloLink-DUB probe for 1 h at 37°C followed by incubation overnight at 4°C. Samples were washed with 350 mM NaCl, 50 mM TrisHCl buffer. The last wash was performed with C3 protease incubation buffer (150 mM NaCl, 50 mM Tris-HCl). The resin was incubated in 100 μL of C3 buffer with 10 μg of C3 protease for 2 h at 4°C with constant shaking. Samples were then spun at 500G in table top centrifuge, supernatant was separated from the beads and incubated for 2 h with 20 μL of GSH resin to remove the C3 protease. Samples were spun at 500G and supernatant was separated from GSH resin. 100 μL of C3 buffer was added to the HaloLink resin and rock for 1 h at 4°C. Supernatant was collected and reduced with 5 mM DTT and alkylated with 10 mM iodoacetamide prior to in-solution tryptic digestion (overnight at 37°C). C18 clean-up was performed and proteins were eluted in 0.1% TNF in water and then dried.

#### Cell culture

Suspension cells (Jurkat) were cultured in RPMI-1640 medium containing 10% fetal bovine serum (FBS), 2mM L-glutamine, 100 units/ml penicillin, 100 μg/ml streptomycin and 50 μM β-mercaptoethanol at 37°C, with 5% CO_2_. Human U2-OS, HeLa S3 (abbreviated to HeLa) and HEK293T cells (abbreviated to 293T), HEK293 cells (abbreviated to 293) were cultured in DMEM (Sigma) containing 10% FBS and penicillin/streptomycin (both Thermo Fisher). To generate doxycycline-inducible cell lines, U2-OS cells stably expressing TetR (Gibbs-Seymour et al., 2016), were transduced with lentivirus generated from pLenti-CMV/TO-Hygro-GFP-ZUFSP^∗siR#2^ WT or pLenti-CMV/TO-Hygro-GFP-ZUFSP^∗siR#2^ C360S constructs and selected in 500 μg/ml hygromycin B (Thermo Fisher). Doxycycline hyclate (Sigma) was added to media at a final concentration of 0.5 μg/ml. Transient DNA transfections were performed with TransIT-LT1 (Mirus) or Polyfect (QIAGEN), and transient siRNA transfections were performed with Lipofectamine RNAiMAX (Thermo Fisher), each according to the manufacturer’s instructions. For depletion of endogenous ZUFSP, optimum silencing occurred after 72 h with two rounds of RNAi transfection within the first 24 h. To generate CRISPR/Cas9-mediated knockout cell lines, HeLa S3 ells were transfected with the PX459 vector containing UBC13-sgRNA. Transfected cells were plated at low density in 1 μg/ml puromycin (Invitrogen). Single colonies were propagated and individual clones were assessed by western blotting. For proteasome inhibition, cells were treated with 20 μM MG-132 (Merck) for 3 h prior to laser micro-irradiation experiments.

#### RNAi and CRISPR/Cas9 sgRNA sequences

The following siRNAs were used in this study:

siCTRL: siGENOME Non-targeting control (Dharmacon)
si*ZUFSP* (#1): AUAUGGAACUUCAGAUAAC
si*ZUFSP* (#2): UUACCAUAUUCUUCAGGAA
si*ZUFSP* (#3): GGUCACAGUCGAACUGUUA
si*ZUFSP* (#4): CAGUCGAACUGUUAUUGGA (Ambion, s48009)
si*ZUFSP* (#5): GGAAGACUGUGAUCAACCA (Ambion, s48010)
si*ZUFSP* (#6): GGAACUUCAGAUAACAAGA (Ambion, s48011)
si*ZUFSP* (#7): GCAGAGACAAUAUGGUUUA; GAUUGGAGCAUGUGAAGUA; CUUCAUAGGUAUUAUCAGA; UACACACCCUCGCUUAUUU (Dharmacon, siGENOME Smartpool)
si*CtIP*: GCUAAAACAGGAACGAAUC

The pSpCas9n(BB)-2A-Puro (PX459) V2.0 vector was a kind gift from Dr Feng Zhang (Addgene plasmid #62988) (Ran et al., 2013). The following sgRNA sequence was used in this study: UBC13-sgRNA: 5′- GGAGGATGGATGGTACCCCCTGG - 3′ (exon 1).

#### Immunopurification of RPA complexes

For co-immunoprecipitation of native complexes, cells were lysed in buffer containing 50 mM Tris-HCl pH 8, 1% Triton X-100, 100 mM NaCl, Benzonase nuclease (Sigma), 1 mM DTT, 5 mM N-Ethylmaleimide (NEM, Sigma), protease and phosphatase inhibitors. Lysates were clarified and added to FLAG-M2 affinity gel for 15 min while rotating at 4°C. Beads were washed several times with lysis buffer and eluted with LDS sample buffer (Thermo Fisher).

#### Nuclear enrichment

Confluent HEK293 cells were washed with PBS and snap-frozen in liquid nitrogen for future usage. Cell pellets were gently resuspended in ice cold buffer A (10 mM HEPES pH 7.9, 1.5 mM MgCl_2_, 10 mM KCl, 1 mM DTT, 1 mM ABSF, 1% PIC (Roche), 1 mM Na_3_VO), kept on ice for 30 min and washed again with buffer A (cells were pelleted at 100 x g, 5 min at 4 C°). Cells were lysed by incubation on ice for 5 mins with 2-3 pellet volumes of buffer A containing 0.1% NP40. Samples were centrifuged at 14000Gfor 10 min at 4°C. Supernatant (cytoplasmic fraction) was collected. The remaining pellet was suspended in 1-2 pellet volumes of buffer C (20 mM HEPES pH 7.9, 1.5 mM MgCl_2_, 84 mM KCl, 1 mM DTT, 1 mM ABSF, 1% protease inhibitor cocktail, 1 mM Na_3_VO, 25% glycerol). Samples were incubated for 30-60 min at 4°C with agitation and centrifuged at 17000 x g for 15 min. Supernatant (nuclear fraction) was collected.

#### Immunoprecipitation for MS analyses

5 μg of anti-ZUFSP antibody or anti-rabbit IgG antibody was conjugated to around 10 μL of magnetic beads (Bio-Rad) for 1 h at 4°C in PBS. Beads were washed with PBS-T (0.1%Tween-20). Nuclear extract from HEK293 cells was prepared using the protocol described above and precleared on anti-rabbit IgG beads for 0.5 h at room temperature. Subsequently 20 mg of precleared lysate was used per immunoprecipitation (IP) with the ZUFSP antibody. IP was carried out for 1 h at room temperature. Both preclearing and ZUFSP conjugated beads were washed 5 times with IP buffer (20 mM HEPES pH 7.9, 1.5 mM MgCl_2_, 84 mM KCl, 1% protease inhibitor cocktail, 5% Glycerol). Proteins were eluted with 120 mM glycine pH 2 (3 time/5 min/30 μl). Acidic pH was neutralised with 1 M Tris-HCl pH 8. Samples were reduced with 5 mM DTT and alkylated with 10 mM IA prior to in-solution tryptic digestion (overnight at 37°C). C18 clean-up was performed, and proteins were eluted in 50% ACN /0.5 TNA and then dried. Preclearing beads were used as a control. The experiment was performed in triplicate.

#### Mass spectrometry

Tryptic peptides were separated by nano-Liquid chromatography (nLC, Dionex ultimate 3000) coupled to mass spectrometry (MS, LTQ-Orbitrap Velos; Thermo scientific) on LC analytical columns with an Acclaim PepMap 100 C_18_ pre-column (Thermo scientific, 100 μm x 2 cm nano Viper, 100 Å, 5 μm, P/N 164564) followed by PepMap RSLC C_18_ peptide separating column (Thermo scientific, 75 μm x 15 cm, 100 Å, 3 μm, P/N ES800) with 120 min gradient using buffer A (0.1% formic acid, 3%DMSO (v/v)) and 1 to 35% of buffer B (80% acetonitrile, 3% DMSO, 0.08% formic acid (v/v)). A parent ion scan was performed in the Orbitrap, using a resolving power of 60000. CID was performed with collision energy 35% and activation time of 10 ms and the top 20 most intense peptides were selected for MS/MS.

#### Protein identification

MS data were analyzed with MaxQuant (1.6.0.13) (Cox and Mann, 2008). Peptides MS/MS spectra were searched against a UniProt complete human sequence database (March 2015). Enzyme specificity was set to trypsin with up to two missed cleavages. Carbamidomethylation was set as a fixed modification while N-term modification was set as variable. MaxQuant LFQ (Label free quantitation) was enabled with match between runs. Proteins were identified with peptide and protein FDR (False Discovery Rate) cut-off of 1%. Spectral count values were extracted and subjected to SAINTexpress (Significance Analysis of INTeractome) (Teo et al., 2014) to identify bona fide ZUFSP interacting proteins. The DAVID functional enrichment tool was used to perform pathway analysis for biological process (Huang et al., 2009).

#### Deubiquitylation assays

DUBs were diluted in 50 mM Tris-HCl pH 7.5, 50 mM NaCl, 10 mM DTT and incubated at 24°C for 10 min. DUB assays were subsequently carried out with 750 ng of tetraUb of different linkage types incubated with 2 μM DUB in a reaction volume of 10 μl. Reactions were incubated at 30°C and stopped at different time points by adding LDS buffer. Samples were separated on a 4%–12% SDS-PAGE gel (Thermo Fisher) and silver stained using Pierce Silver stain kit (Thermo Fisher).

#### UBD linkage specificity analysis

TetraUb chains of the different linkage types were assembled and purified as described previously (Kristariyanto et al., 2015a, Kristariyanto et al., 2015b). Halo-tagged ZUFSP domains used in this study (10.5 mmol) were incubated with 100 μl of HaloLink resin (Promega) in 500 μl of coupling buffer (50 mM Tris-HCl pH 7.5, 150 mM NaCl, 0.05% NP-40, 1 mM DTT) for 3 h at 4°C. UBD linkage specificity analysis was carried out by incubating 10 μl of the coupled Halo-fusion protein resin with 1 μg of tetraUb of the indicated linkages in pull-down buffer (50 mM Tris-HCl pH 7.5, 150 mM NaCl, 0.1% NP-40, 1 mM DTT, 0.5 mg/ml BSA) for 2 h at 4°C. The resin was washed two times with wash buffer (50 mM Tris-HCl pH 7.5, 250 mM NaCl, 0.2% NP-40, 1 mM DTT) and once with coupling buffer. Captured tetraUb chains were eluted by adding LDS buffer, separated on a 4%–12% SDS-PAGE gel, and visualized by silver staining.

For analysis of ZUFSP Ub binding from cells, 1 mg of HEK293 cell lysate was incubated with 10 μL Halo-UBD resin for 2 h at 4°C. Beads were washed with lysis buffer containing 150mM NaCl and eluted with 2X LDS (Thermo Fisher) and bound polyUbs were analyzed by immunoblotting.

#### Isothermal titration calorimetry (ITC)

ITC measurements were performed on a MicroCal PEAQ-ITC (Malvern) at 25°C with a setting of 20 × 2 μl injections. Proteins were dialyzed into 50 mM HEPES pH 7.5, 150 mM NaCl, and 250 μM TCEP. For indicated measurements, the syringe contained truncated ZUFSP proteins at concentration of 320 μM, and the cell contained polyUbs at a concentration of 16 μM. For indicated measurements, the syringe contained ZUFSP UBD at concentration of 260 μM, and the cell contained polyUbs at a concentration of 16 μM.

#### Immunofluorescence, laser micro-irradiation and microscopy

Immunofluorescence protocols were as described previously. Laser micro-irradiation was performed as described previously (Gibbs-Seymour et al., 2016). Briefly, cells were pre-sensitized for 24 h before laser micro-irradiation using 10 μM BrdU (Sigma) and the media was changed to fully supplemented phenol-red free DMEM plus HEPES (Thermo Fisher) before laser micro-irradiation and imaging. Laser micro-irradiation was performed on an Olympus Fluoview FV1200 confocal microscope equipped an inverted IX83 motorised stage with a 37°C humidified chamber and 60x/1.40 oil UPlanSApo objective and 405nm laser. Confocal microscopy was performed on the same microscope. Standard wide-field microscopy was performed on the Olympus BX61 microscope system, equipped with 20x/0.5 and 40x/0.75 dry objectives, a CoolSNAP HQ2 camera (Roper Scientific) and MetaMorph 7.5 imaging software (GE Healthcare).

#### Quantitative image-based cytometry (QIBC)

QIBC was performed essentially as described (Toledo et al., 2013, Ochs et al., 2016). For the chromatin-bound analysis of γH2AX and 53BP1, cells were pre-extracted with 0.2% Triton X-100 for 5 mins on ice before fixation and subsequent processing for immunostaining. For 53BP1 and cyclin A analysis, the pre-extraction step was omitted. Images were randomly acquired across technical duplicates under non-saturating conditions for the sample exhibiting the highest signal intensity and the settings subsequently applied to all other samples. Images were acquired with the Olympus BX61 microscope system described above using the 20x/0.5 objective. Typically, 20-25 non-overlapping images were randomly acquired per condition using a motorised stage (Photometrics), yielding 2000-3500 single cells. Image analysis was performed using custom pipelines in CellProfiler (Carpenter et al., 2006). The resulting data was then exported and processed in Spotfire (Tibco) software.

#### Clonogenic assays

For colony formation assays, U2-OS cells were transfected with siRNAs, and then plated at low densities in the presence of the indicated doses of camptothecin (CPT) or alternatively plated and then treated the next day with the indicated dose of ionising radiation (IR). Cells were subsequently fixed and stained with crystal violet after 12 days. The surviving fraction at each dose was calculated after normalization to the plating efficiency of untreated samples.

### Quantitation and Statistical Analysis

SDS gels of DUB assays used for gel-based quantification were stained overnight with Sypro Ruby and scanned with the Gel Doc XR+ and quantified with ImageStudioLite. Data from three independent experiments were fitted using nonlinear regression, one phase exponential decay. SD error bars are shown.

Qualitative microscopy analysis is representative of between 3-7 independent biological replicates. For quantitative assessment of 53BP1 foci positivity, data represents mean ± SEM from two biologically independent experiments using technical duplicates per datapoint. For analysis of cell survival after clastogen exposure, data represents mean ± SEM from two biologically independent experiments using technical triplicates per datapoint.

### Data and Software Availability

The accession number for the crystal structures of the ZUFSP∼UbPrg complex reported in this paper is PDB: 6FGE.

The accession number for the mass spectrometry proteomics data reported in this paper is ProteomeXchange Consortium via the PRIDE (Vizcaíno et al., 2016) partner repository: PXD008509.

## Supplemental Information

Document S1. Figures S1–S7 and Table S1

Document S2. Article plus Supplemental Information
