# Supplementary material for: Discovery and Characterization of ZUFSP/ZUP1, a Distinct Deubiquitinase Class Important for Genome Stability
Source: Mol Cell. 2018 Apr 5;70(1):150–164.e6. doi: 10.1016/j.molcel.2018.02.023 (PMC5896202; doi:10.1016/j.molcel.2018.02.023)
Supplement: Document S1. Figures S1–S7 and Table S1 [file mmc1.pdf]

Molecular Cell, Volume *70*

## Supplemental Information

# Discovery and Characterization of ZUFSP/ZUP1, a Distinct Deubiquitinase Class Important for Genome Stability

**Dominika Kwasna, Syed Arif Abdul Rehman, Jayaprakash Natarajan, Stephen Matthews, Ross Madden, Virginia De Cesare, Simone Weidlich, Satpal Virdee, Ivan Ahel, Ian Gibbs-Seymour, and Yogesh Kulathu**

# Supplementary Figure Legends

## Figure S1

**A**

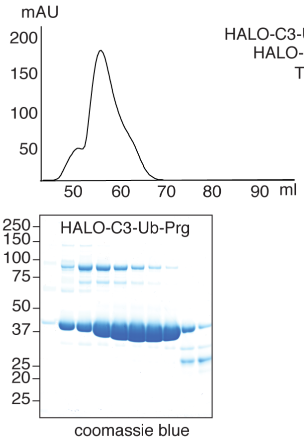


**B**

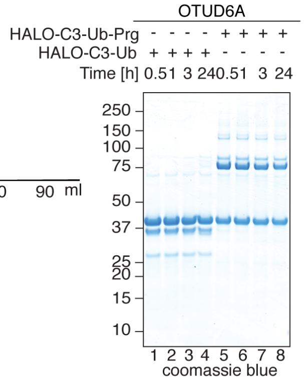


**C**

| NR | DUB | Mass | JURKAT | HEK293 | DUB FAMILY |
|---|---|---|---|---|---|
| 1 | USP9X | 292 kDa | + | + | USP |
| 2 | USP24 | 294 kDa | + | + | |
| 3 | USP7 | 128 kDa | + | + | |
| 4 | USP47 | 157 kDa | + | + | |
| 5 | USP11 | 110 kDa | + | + | |
| 6 | USP5 | 96 kDa | + | + | |
| 7 | USP19 | 146 kDa | + | + | |
| 8 | USP14 | 56 kDa | + | + | |
| 9 | USP25 | 122 kDa | + | + | |
| 10 | USP32 | 182 kDa | + | + | |
| 11 | USP8 | 128 kDa | + | + | |
| 12 | USP4 | 109 kDa | + | + | |
| 13 | UBP48 | 119 kDa | + | + | |
| 14 | UBP34 | 404 kDa | + | + | |
| 15 | UBP15 | 112 kDa | + | + | |
| 16 | UBP13 | 97 kDa | + | + | |
| 17 | UBP10 | 87 kDa | + | + | |
| 18 | UBP28 | 122 kDa | + | + | |
| 19 | UBP37 | 110 kDa | + | + | |
| 20 | UBP38 | 117 kDa | + | + | |
| 21 | UBP16 | 94 kDa | + | + | |
| 22 | CYLD | 107 kDa | + | + | |
| 23 | UBP20 | 102 kDa | + | + | |
| 24 | UBP42 | 145 kDa | + | + | |
| 25 | UBP40 | 140 kDa | + | + | |
| 26 | UBP3 | 59 kDa | + | + | |
| 27 | UBP33 | 107 kDa | + | + | |
| 28 | UBP22 | 60 kDa | + | + | |
| 29 | UBP30 | 59 kDa | + | + | |
| 30 | UBP45 | 92 kDa | + | + | |
| 31 | UBP35 | 113 kDa | + | + | |
| 32 | UBP6 | 159 kDa | + | + | |
| 33 | UBP36 | 123 kDa | + | + | |
| 34 | UBP27 | 50 kDa | + | + | |
| 35 | UBP53 | 121 kDa | - | + | |
| 36 | UBP31 | 147 kDa | - | + | |
| 37 | UBP54 | 187 kDa | - | + | |
| 38 | UBP2 | 68 kDa | - | + | |
| 39 | UBP46 | 42 kDa | - | + | |
| 40 | VCIP1 | 134 kDa | + | + | OTU |
| 41 | OTUD4 | 124 kDa | + | + | |
| 42 | OTU7B | 93 kDa | + | + | |
| 43 | OTUD5 | 61 kDa | + | + | |
| 44 | OTU1 | 38 kDa | + | + | |
| 45 | OTU6B | 34 kDa | + | + | |
| 46 | OTUD3 | 45 kDa | + | + | |
| 47 | OTUB2 | 27 kDa | + | + | |
| 48 | OTU7A | 101 kDa | + | + | |
| 49 | OTUD1 | 51 kDa | + | + | |
| 50 | OTULIN | 40 kDa | + | - | |
| 51 | OTUB1 | 31 kDa | - | + | |
| 52 | UCHL5 | 38 kDa | + | + | UCHL |
| 53 | BAP1 | 80 kDa | + | + | |
| 54 | UCHL3 | 26 kDa | + | + | |
| 55 | UCHL1 | 25 kDa | - | + | |
| 56 | FAM63B | 67 kDa | + | + | MINDY |
| 57 | FAM188A | 50 kDa | + | + | |
| 58 | FAM188B | 84 kDa | + | + | |
| 59 | FAM63A | 52 kDa | - | + | |
| 60 | ATX3 | 42 kDa | + | + | MJD |
| 61 | JOSD1 | 23 kDa | + | + | |
| 62 | AT7L3 | 39 kDa | + | + | |
| 63 | JOSD2 | 21 kDa | + | + | |
| 64 | ZUFSP | 66 kDa | + | + | ? |

**D**

| SEQUENCE | INTENSITY HEK293 HALO-C3-UB-PRG | START POSITION | END POSITION |
|---|---|---|---|
| MLSCNICGETVTSEPDMK | 1268600 | 1 | 18 |
| HEGFYSENLTESR | 1444800 | 115 | 127 |
| GSVYETTYSPPECPFCGK | 2055500 | 144 | 161 |
| QEIEEFQK | 2527400 | 255 | 262 |
| EGFDPQGASQLNNR | 1716200 | 403 | 416 |

| SEQUENCE | INTENSITY JURKAT HALO-C3-UB-PRG | START POSITION | END POSITION |
|---|---|---|---|
| MLSCNICGETVTSEPDMK | 238550 | 1 | 18 |
| INTVQYGTSDNK | 918930 | 65 | 76 |
| DNTLQCGMEVNSSILSGCASNHPK | 5510200 | 78 | 101 |
| GSVYETTYSPPECPFCGK | 1817500 | 144 | 161 |
| IEEHSEDMETHVK | 9126500 | 162 | 174 |
| VQCSGDLQLAHQLQQEEDR | 3988200 | 228 | 246 |
| QYGLDNSGGYK | 2433400 | 266 | 276 |
| EGFDPQGASQLNNR | 1474700 | 403 | 416 |

**E**

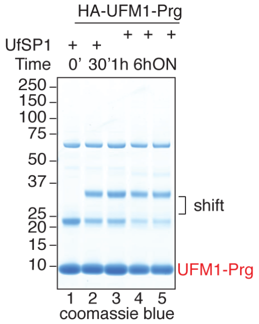

**Figure S1 (related to Figure 1).**

**Activity based protein profiling of DUBs identifies ZUFSP as a putative DUB**

**(A)** Protein profile and Coomassie Blue stained gel of HALO-C3-Ub-Prg probe after purification by size exclusion chromatography.

**(B)** Analysis of probe activity by incubating HALO-C3-Ub-Prg and HALO-C3-Ub with the well characterised DUB OTUD6A. Samples were separated by SDS-PAGE gel and visualised by Coomassie Blue staining.

**(C)** Full list of the annotated DUBs discovered by mass spectrometry analysis from the Ub-Prg probe after incubation with HEK293 and JURKAT cells. DUB family names are shown in the last column.

**(D)** Details of ZUFSP peptides identified by mass spectrometry analysis following Ub-Prg probe pull downs from HEK293 or JURKAT cell extracts.

(**E**) Reaction of HA-UFM1-Prg probe with UfSP1 (Mm). Samples were separated by SDS-PAGE gel and visualised by Coomassie Blue staining.

**Figure S2**

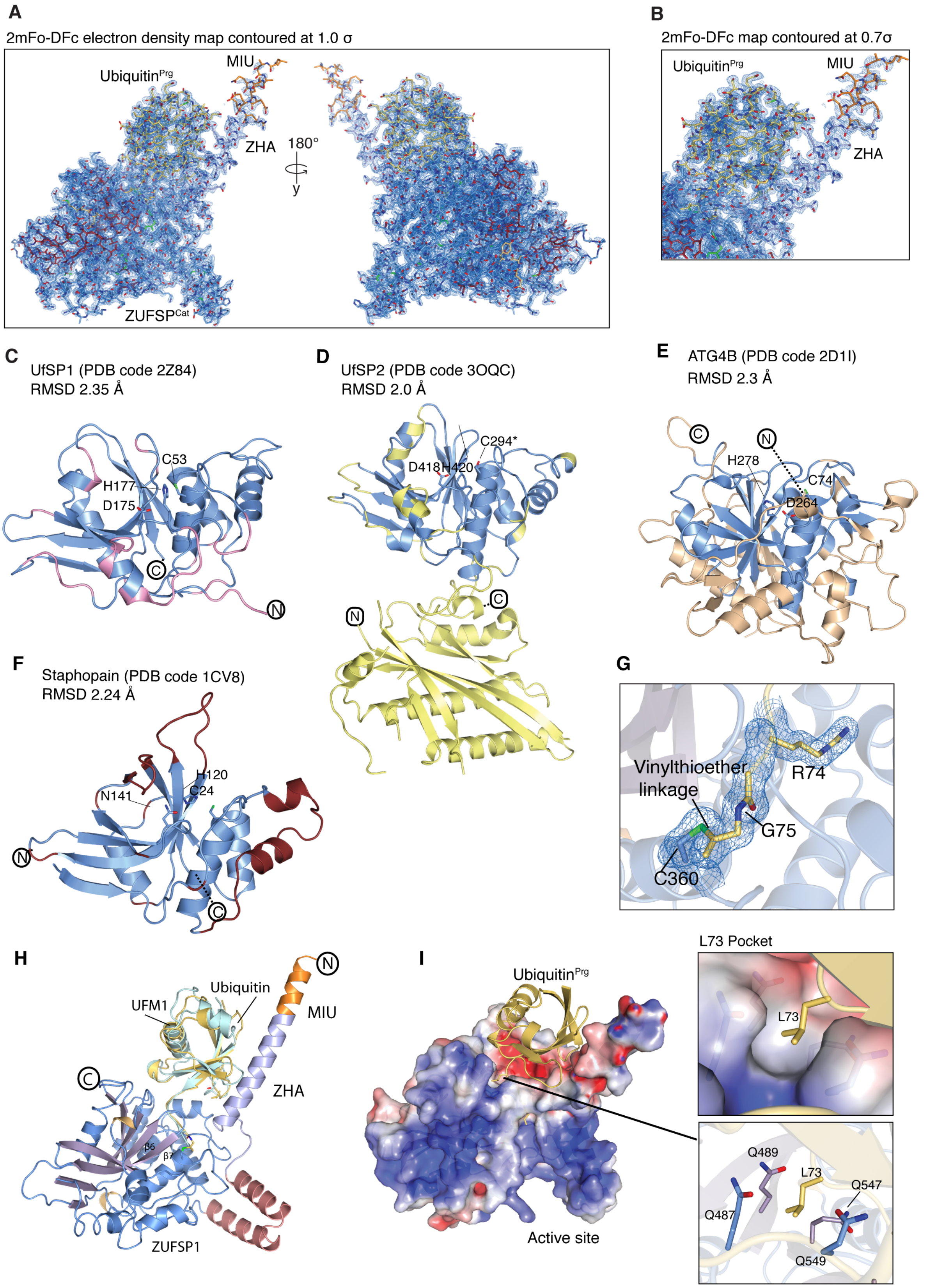

A
2mFo-DFc electron density map contoured at 1.0 σ
Ubiquitin^Prg
MIU
ZHA
180°
y
ZUFSP^Cat
B
2mFo-DFc map contoured at 0.7σ
Ubiquitin^Prg
MIU
ZHA
C
UfSP1 (PDB code 2Z84)
RMSD 2.35 Å
C53
H177
D175
C
N
D
UfSP2 (PDB code 3OQC)
RMSD 2.0 Å
C294*
D418
H420
N
C
E
ATG4B (PDB code 2D1I)
RMSD 2.3 Å
C
N
H278
C74
D264
F
Staphopain (PDB code 1CV8)
RMSD 2.24 Å
H120
C24
N141
N
C
G
Vinylthioether
linkage
R74
G75
C360
H
UFM1
Ubiquitin
N
MIU
ZHA
C
β6
β7
ZUFSP1
I
Ubiquitin^Prg
Active site
L73 Pocket
L73
Q489
L73
Q547
Q487
Q549

**Figure S2 (related to Figure 2).**

**Crystal structure of ZUFSP in complex with distal ubiquitin**

(**A**) 2|Fo|- |Fc| electron density map of the asymmetric unit for the ZUFSP~Ub-Prg complex contoured at 1σ.

**(B)** 2|Fo|- |Fc| electron density map of the ZHA-MIU region contoured at 0.7σ. The electron density for the residues from 236 till 239 is poor and the residues in this range have been built at 50% occupancy.

(**C-F**) Superposition of ZUFSP with UFSP1, UFSP2, ATG4B and Staphopain. The aligned regions of these structures are depicted in blue. The structural alignment was done in coot using Secondary Structure Matching (SSM) superposition method.

**(G)** Weighted 2|F0|-|Fc| electron density map of catalytic cysteine in covalent linkage with ubiquitin-Prg contoured at 1σ.

**(H)** Structural superposition of UFM1 onto the ZUFSP-Ub-Prg complex structure. Ubiquitin and UFM1 are coloured in yelloworange and lightcyan, respectively.

**(I)** Close up view of the L73 pocket formed by the aliphatic side chains of glutamine residues is shown both in surface representation and sticks. Negative electrostatic surface is shown in red and positive surface in blue and hydrophobic in white. The surfaces are drawn at ±4e/kBT

Figure S3

A

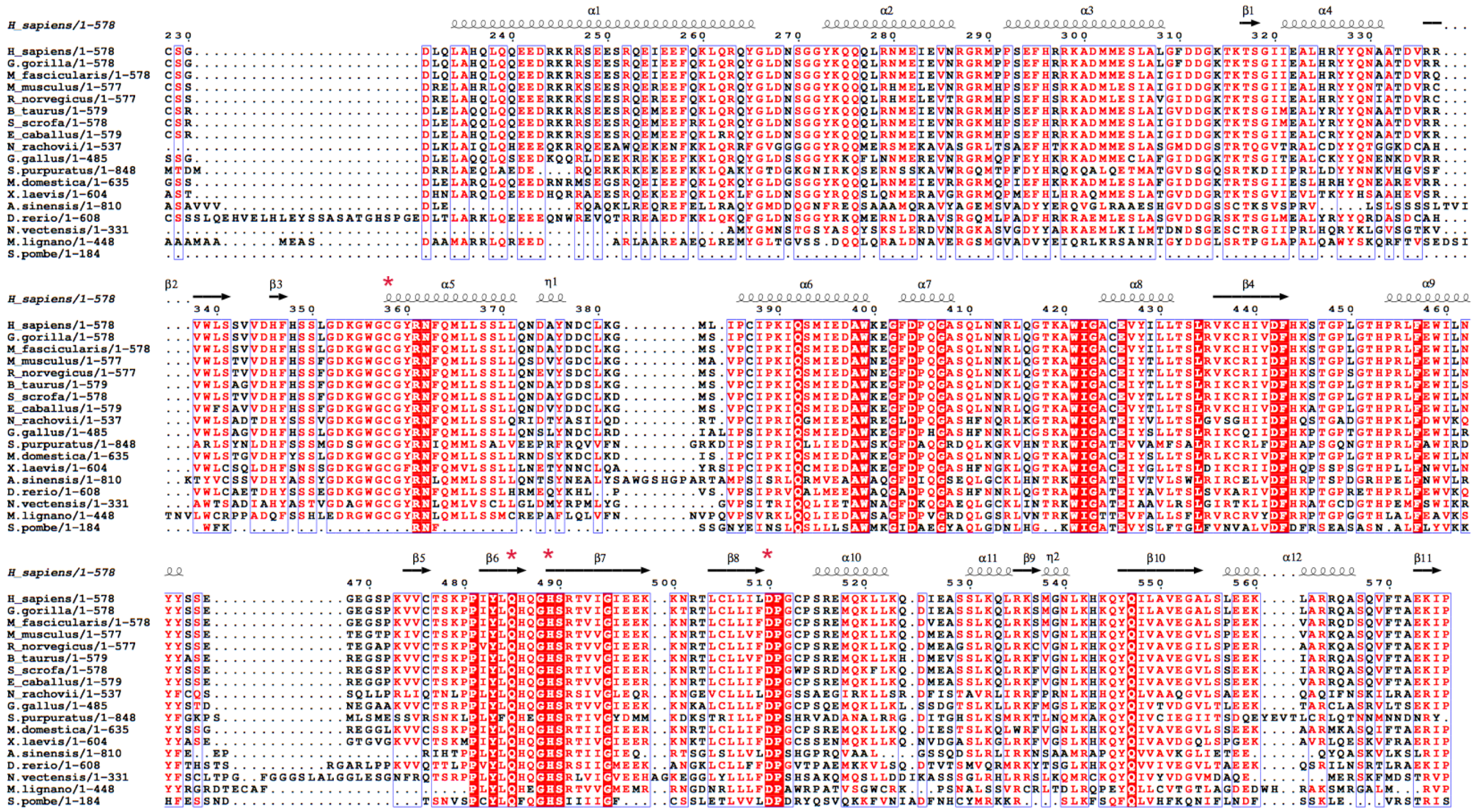

**Figure S3 (related to Figure 2,3).**

**ZUFSP is evolutionary conserved.**

Sequence alignment of the catalytic domain of ZUFSP from different species (human, gorilla, monkey, mouse, rat, bovine, pig, horse, fish, chicken, sea urchin, fly, frog, alligator, zebrafish, sea anemone, flatworm, yeast). The region covering the catalytic domain shows relatively higher degree of evolutionary conservation. Fully conserved residues are shaded in red. Secondary structure elements are represented by coil and arrows for alpha helix and beta sheets respectively. The catalytic residues are highlighted in red asterisks.

## Figure S4

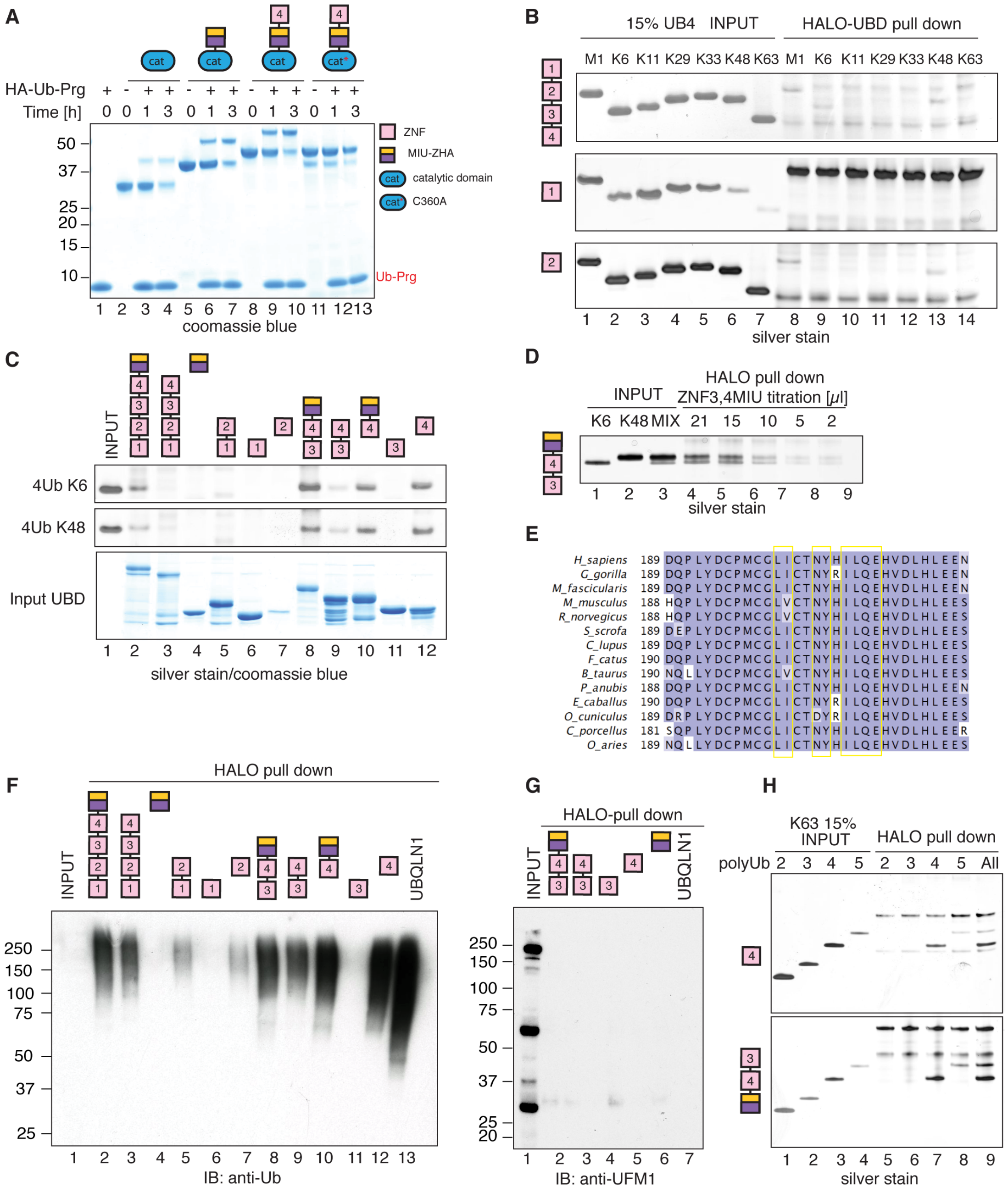
A
HA-Ub-Prg
Time [h]
ZNF
MIU-ZHA
catalytic domain
C360A
Ub-Prg
coomassie blue
B
15% UB4 INPUT
HALO-UBD pull down
M1 K6 K11 K29 K33 K48 K63 M1 K6 K11 K29 K33 K48 K63
silver stain
C
INPUT
4Ub K6
4Ub K48
Input UBD
silver stain/coomassie blue
D
INPUT
HALO pull down
ZNF3,4MIU titration [μl]
K6 K48 MIX 21 15 10 5 2
silver stain
E
H_sapiens
G_gorilla
M_fascicularis
M_musculus
R_norvegicus
S_scrofa
C_lupus
F_catus
B_taurus
P_anubis
E_caballus
O_cuniculus
C_porcellus
O_aries
F
HALO pull down
INPUT
UBQLN1
IB: anti-Ub
G
HALO-pull down
INPUT
UBQLN1
IB: anti-UFM1
H
K63 15% INPUT
HALO pull down
polyUb 2 3 4 5 2 3 4 5 All
silver stain

**Figure S4 (related to Figure 3).**

**Characterization of ubiquitin binding domains in ZUFSP**

**(A)** The indicated recombinant ZUFSP proteins were incubated with the HA-Ub-Prg probe. Reactions were separated by SDS-PAGE and visualised by Coomassie Blue staining.

**(B)** HALO-tag proteins were immobilised on HALO Link resin and incubated with tetra-Ub of different linkages. Bound material was separated by SDS-PAGE and silver stained.

**(C)** Comparison of polyUb binding by different domains of ZUFSP. Bound material was analysed as in (B). In the bottom panel, 5 % of input UBD stained by Coomassie Blue is shown.

**(D)** Competitive binding assay of K6 and K48 4Ub by HALO-ZUFSP ZNF3,4MIU (145-267). Bound material was analysed as in (B).

**(E)** Multiple sequence alignment of ZNF4 (189-219) of ZUFSP from different species. Yellow boxes highlight residues mutated, and tested for binding ability, shown in **Figure 4F**.

**(F-G)** PolyUb chains isolated from mammalian cells with recombinant domains of ZUFSP. HEK293 cell lysate was incubated with the indicated domains immobilised to HALO-tag resin. Bound material was visualised by anti-Ub and anti-UFM1 immunoblotting.

**(H)** The indicated ZUFSP HALO-Tag fusion proteins were coupled to HALO Link beads and incubated with di-, tri-, tetra-, or penta-K63Ub. The last well shows competitive pull down with di-, tri-, tetra- and penta-Ub mixed together. Bound material was analysed as in (B).

# Figure S5

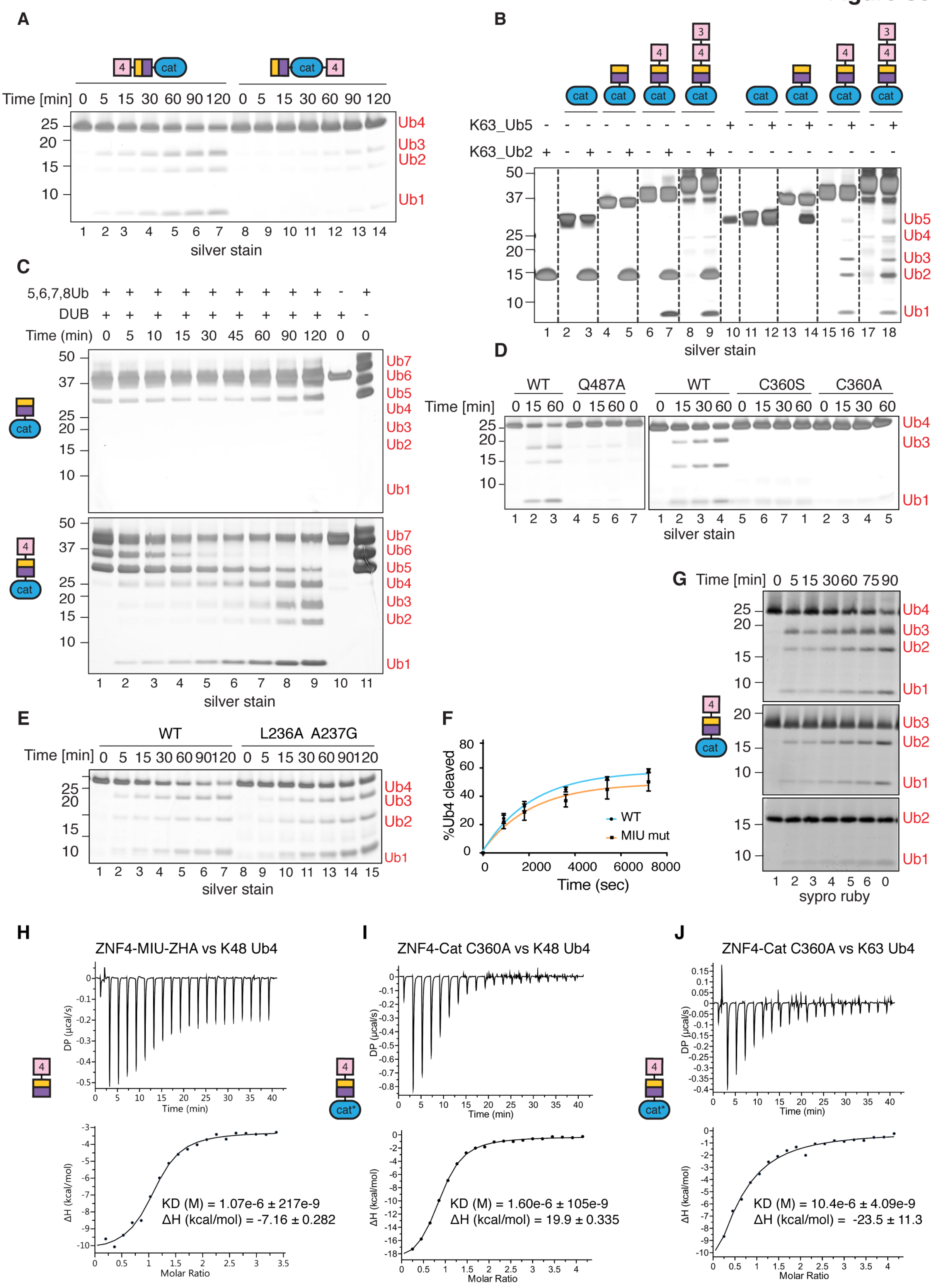

A
Time [min] 0 5 15 30 60 90 120 0 5 15 30 60 90 120
Ub4
Ub3
Ub2
Ub1
silver stain
B
K63_Ub5
K63_Ub2
Ub5
Ub4
Ub3
Ub2
Ub1
silver stain
C
5,6,7,8Ub
DUB
Time (min) 0 5 10 15 30 45 60 90 120 0 0
Ub7
Ub6
Ub5
Ub4
Ub3
Ub2
Ub1
silver stain
D
WT
Q487A
C360S
C360A
Time [min]
silver stain
E
WT
L236A A237G
Time [min] 0 5 15 30 60 90 120 0 5 15 30 60 90 120
silver stain
F
%Ub4 cleaved
Time (sec)
WT
MIU mut
G
Time [min] 0 5 15 30 60 75 90
sypro ruby
H
ZNF4-MIU-ZHA vs K48 Ub4
I
ZNF4-Cat C360A vs K48 Ub4
J
ZNF4-Cat C360A vs K63 Ub4
DP (µcal/s)
Time (min)
ΔH (kcal/mol)
Molar Ratio
KD (M) = 1.07e-6 ± 217e-9
ΔH (kcal/mol) = -7.16 ± 0.282
KD (M) = 1.60e-6 ± 105e-9
ΔH (kcal/mol) = 19.9 ± 0.335
KD (M) = 10.4e-6 ± 4.09e-9
ΔH (kcal/mol) = -23.5 ± 11.3

**Figure S5 (related to Figure 5).**

**Analysis of DUB activity of ZUFSP**

**(A)** Deubiquitinase assay monitoring activity of $ZUFSP^{ZNF4\text{-}MIU\text{-}Cat}$ and $ZUFSP^{MIU\text{-}Cat\text{-}ZNF4}$. The enzymes were incubated with K63-tetraUb for the indicated times. Reaction products were separated by SDS-PAGE and visualized by silver staining.

**(B)** Cleavage of K63-linked pentaUb chains by the indicated ZUFSP constructs. Reaction products were visualized by silver staining.

**(C)** Activity of ZUFSP towards long K63-linked chains was analyzed in a time course monitoring cleavage of mixed penta-, hexa- and hepta-K63-linked poly-Ub chains by $ZUFSP^{MIU\text{-}Cat}$ or $ZUFSP^{ZNF4\text{-}MIU\text{-}Cat}$. Reaction products were visualised by silver staining.

**(D)** Analysis of Q487A mutation on DUB activity and comparison of mutation of catalytic cysteine to serine or alanine. Reaction products were visualized by silver staining.

**(E)** Comparison of $ZUFSP^{ZNF4MIU\text{-}Cat}$ WT with double point mutant in the MIU motif (L263A, A237G). Time course examining cleavage of K63-linked tetraUb. Reaction products were separated by SDS-PAGE and visualized by silver staining.

**(F)** Time course comparing activity of $ZUFSP^{ZNF4\text{-}MIU\text{-}Cat}$ WT and the MIU mutant (L263A, A237G). Percentage of cleaved tetra-ubiquitin was quantified from Sypro Ruby-stained gels. Data from three independent experiments were fitted using nonlinear regression, one phase exponential decay. SD error bars are shown.

**(G)** Time course comparing cleavage of K63 chains of different lengths by $ZUFSP^{ZNF4\text{-}MIU\text{-}Cat}$. Reaction products were separated by SDS-PAGE and visualized by Sypro Ruby staining.

**(H-J)** ITC measurements. **(H)** ZNF4-MIU binding to K48-linked tetraUb **(I)** $ZUFSP^{ZNF4\text{-}MIU\text{-}Cat\ C360A}$ binding to K48-linked tetraUb **(J)** $ZUFSP^{ZNF4\text{-}MIU\text{-}Cat\ C360A}$ binding to K63-linked tetraUb. $K_d$ and $\Delta H$ values of each measurement are indicated.

# Figure S6

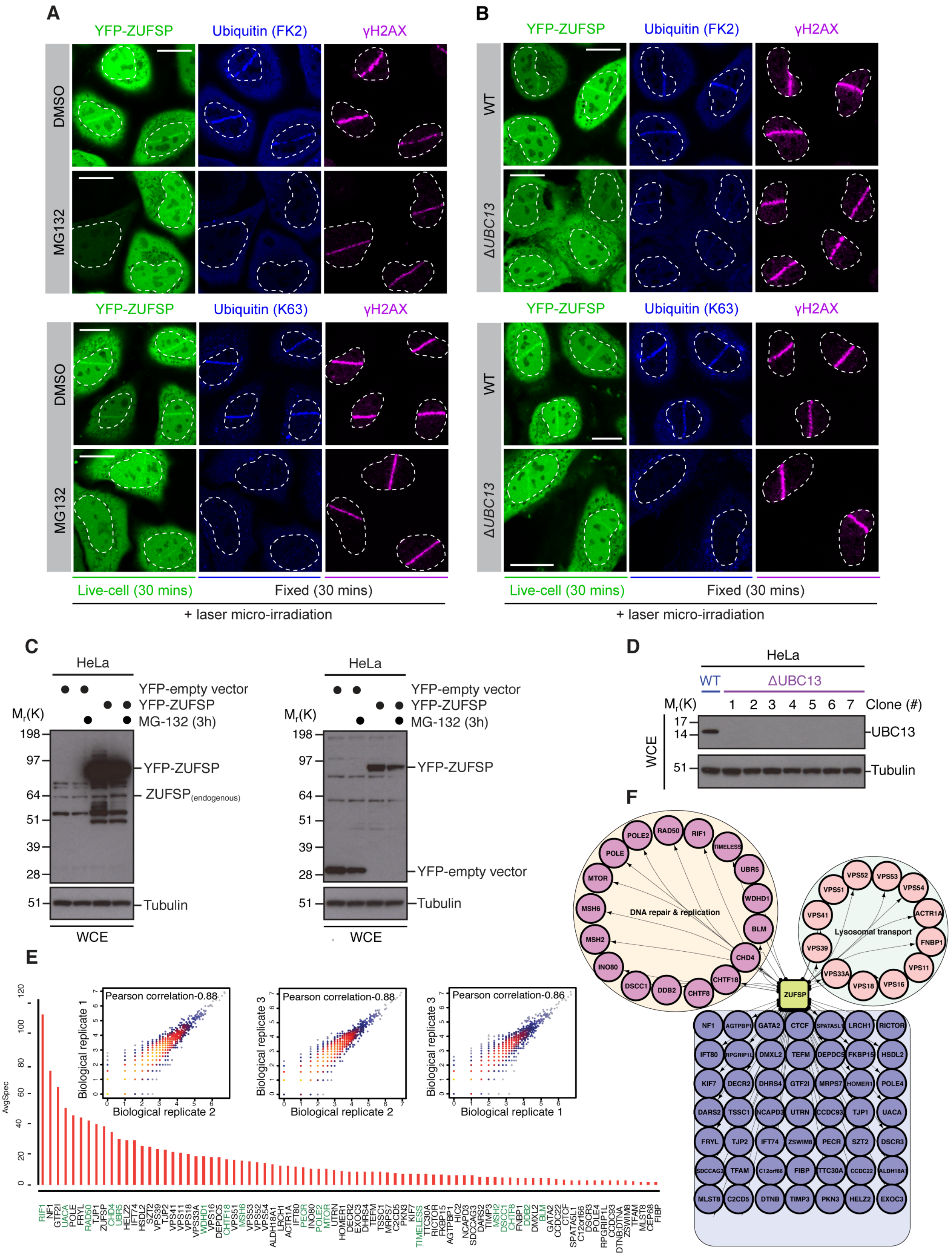

A
B
YFP-ZUFSP
Ubiquitin (FK2)
γH2AX
Ubiquitin (K63)
DMSO
MG132
WT
ΔUBC13
Live-cell (30 mins)
Fixed (30 mins)
+ laser micro-irradiation
C
HeLa
YFP-empty vector
YFP-ZUFSP
MG-132 (3h)
Mr(K)
198
97
64
51
39
28
ZUFSP(endogenous)
Tubulin
WCE
D
WT
ΔUBC13
Clone (#)
1 2 3 4 5 6 7
17
14
UBC13
WCE
E
AvgSpec
Pearson correlation-0.88
Pearson correlation-0.86
Biological replicate 1
Biological replicate 2
Biological replicate 3
F
DNA repair & replication
Lysosomal transport
ZUFSP
POLE2
RAD50
RIF1
TIMELESS
POLE
UBR5
MTOR
WDHD1
MSH6
BLM
MSH2
CHD4
INO80
CHTF18
DSCC1
DDB2
CHTF8
VPS52
VPS53
VPS51
VPS54
VPS41
ACTR1A
FNBP1
VPS39
VPS11
VPS33A
VPS18
VPS16
NF1
AGTPBP1
GATA2
CTCF
SPATA5L1
LRCH1
RICTOR
IFT80
RPGRIP1L
DMXL2
TEFM
DEPDC5
FKBP15
HSDL2
KIF7
DECR2
DHRS4
GTF2I
MRPS7
HOMER1
POLE4
DARS2
TSSC1
NCAPD3
UTRN
CCDC93
TJP1
UACA
FRYL
TJP2
IFT74
ZSWIM8
PECR
SZT2
DSCR3
SDCCAG3
TFAM
C12orf66
FIBP
TTC30A
CCDC22
ALDH18A1
MLST8
C2CD5
DTNB
TIMP3
PKN3
HELZ2
EXOC3

**Figure S6 (related to Figure 6).**

**Analysis of the ZUFSP interactome and the impact of ubiquitin signaling on ZUFSP recruitment to DNA lesions.**

**(A)** HeLa cells were transfected with YFP-ZUFSP, treated with DMSO or MG132 (3h) and then subjected to laser micro-irradiation and imaged at the indicated time. Cells were subsequently fixed and processed for immunostaining with the indicated antibodies. Scale bar, 10 μm.

**(B)** HeLa wild type (WT) or UBC13-null (*ΔUBC13*) cells were transfected with YFP-ZUFSP, subjected to laser micro-irradiation and then imaged at the indicated time. Cells were subsequently fixed and processed for immunostaining with the indicated antibodies. Scale bar, 10 μm.

**(C)** HeLa cells were transfected with either YFP-empty vector or YFP-ZUFSP, treated with DMSO or MG132 (3h), lysed and then analysed by immunoblotting with the indicated antibodies.

**(D)** HeLa wild type (WT) and multiple Δ*UBC13* clones were lysed and analysed by immunoblotting with the indicated antibodies.

**(E)** SAINT (Significance Analysis of INTeractome) analysis of ZUFSP endogenous immunoprecipitation. Pearson correlation of spectral count values between three biological replicates. In the bottom panel histogram presenting the abundance of interacting proteins is shown. DNA repair and replication proteins are highlighted green.

**(F)** ZUFSP interaction map, representing high confidence interacting proteins obtained from SAINT output filtered with 1% Bayesian false discovery rate (BFDR). Proteins were clustered according to DAVID GOBP enrichment.

# Figure S7

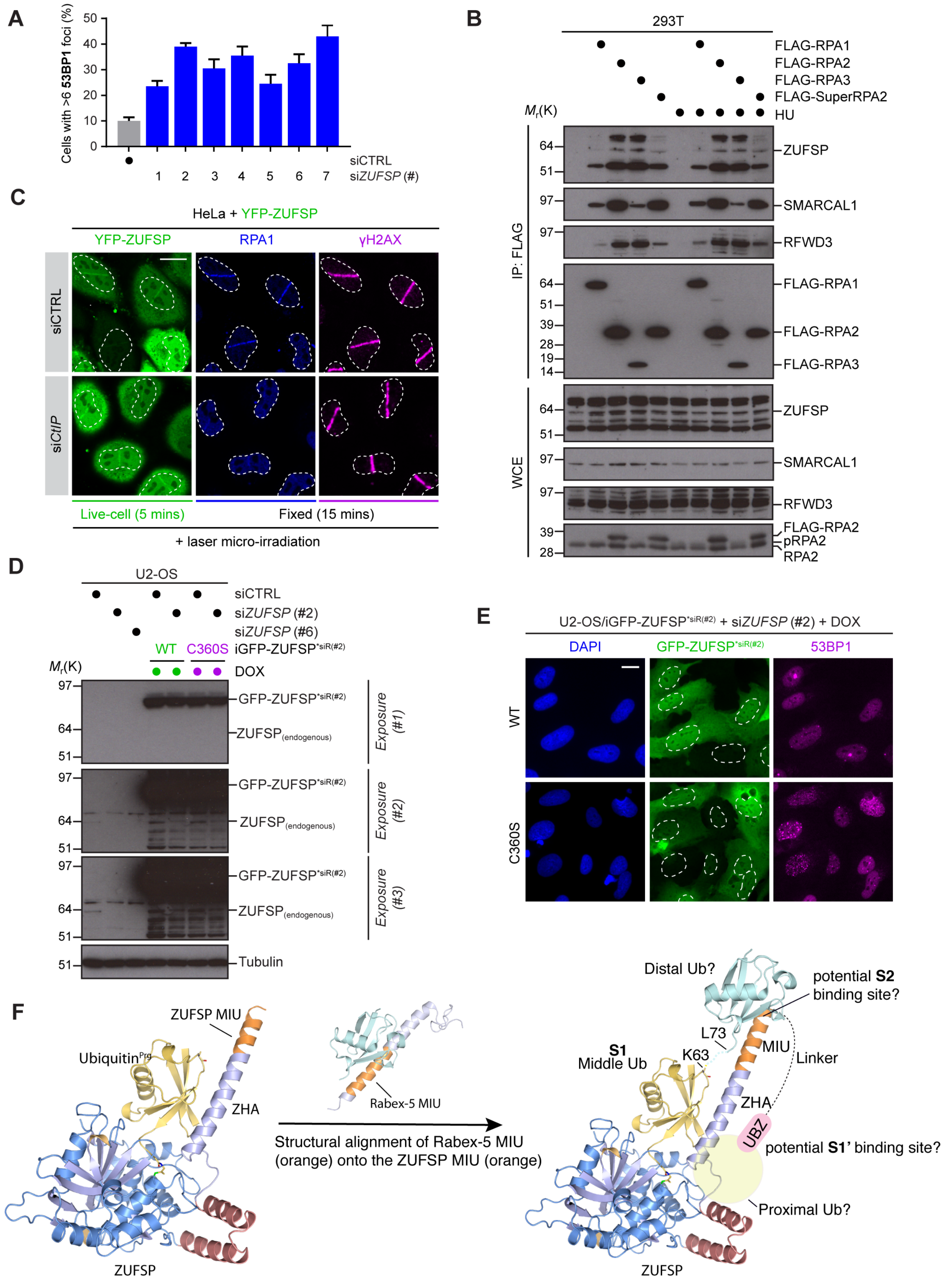
A
Cells with >6 53BP1 foci (%)
50
40
30
20
10
0
siCTRL
siZUFSP (#)
1 2 3 4 5 6 7
B
293T
FLAG-RPA1
FLAG-RPA2
FLAG-RPA3
FLAG-SuperRPA2
HU
Mr(K)
IP: FLAG
WCE
ZUFSP
SMARCAL1
RFWD3
FLAG-RPA1
FLAG-RPA2
FLAG-RPA3
FLAG-RPA2
pRPA2
RPA2
C
HeLa + YFP-ZUFSP
YFP-ZUFSP
RPA1
γH2AX
siCTRL
siCtIP
Live-cell (5 mins)
Fixed (15 mins)
+ laser micro-irradiation
D
U2-OS
siCTRL
siZUFSP (#2)
siZUFSP (#6)
WT
C360S
iGFP-ZUFSP*siR(#2)
DOX
GFP-ZUFSP*siR(#2)
ZUFSP(endogenous)
Exposure (#1)
Exposure (#2)
Exposure (#3)
Tubulin
E
U2-OS/iGFP-ZUFSP*siR(#2) + siZUFSP (#2) + DOX
DAPI
GFP-ZUFSP*siR(#2)
53BP1
WT
C360S
F
ZUFSP MIU
Ubiquitin^Prg
ZHA
ZUFSP
Rabex-5 MIU
Structural alignment of Rabex-5 MIU (orange) onto the ZUFSP MIU (orange)
Distal Ub?
potential S2 binding site?
L73
S1
Middle Ub
K63
MIU
Linker
ZHA
UBZ
potential S1' binding site?
Proximal Ub?
ZUFSP

**Figure S7 (related to Figure 7).**

**ZUFSP is functionally involved in DNA repair**

**(A)** U2-OS cells were transfected with control (siCTRL) or ZUFSP (si*ZUFSP*) siRNAs for 72 h. Cells were processed for immunostaining and enumerated for 53BP1 positivity. Data represents mean ± SEM from two biologically independent experiments using technical duplicates per datapoint.

**(B)** 293T cells were transfected with FLAG-empty vector, FLAG-RPA1, FLAG-RPA2, FLAG-RPA3 or FLAG-SuperRPA (untagged RPA1, FLAG-RPA2, untagged-RPA3) and treated with hydroxyurea (4 h, 2 mM). Cells were lysed, subjected to FLAG immunoprecipitation and analysed by immunoblotting with the indicated antibodies.

**(C)** HeLa cells were transfected with control (siCTRL) or CtIP (si*CtIP*) siRNAs and then further transfected with YFP-ZUFSP. Cells were then subjected to laser micro-irradiation and imaged at the indicated time, fixed and processed for immunostaining with the indicated antibodies. Scale bar, 10 μm.

**(D)** U2-OS, U2-OS/iGFP-ZUFSP$^{*siR(\#2)}$ wild type (WT) or U2-OS/iGFP-ZUFSP$^{*siR(\#2)}$ C360S cells were transfected with either control (siCTRL) or ZUFSP (si*ZUFSP*) siRNAs for 72 h with or without addition of doxycycline. Cells were then lysed and analysed by immunoblotting with the indicated antibodies.

**(E)** U2-OS/iGFP-ZUFSP$^{*siR(\#2)}$ wild type (WT) or U2-OS/iGFP-ZUFSP$^{*siR(\#2)}$ C360S cells were transfected with ZUFSP (si*ZUFSP*) siRNA for 72 h with or without addition of doxycycline. Cells were fixed and processed for immunostaining with the indicated antibodies. Enumeration of 53BP1 foci is shown in Figure 7F. Scale bar, 10 μm.

**(F)** Model depicting substrate binding and catalysis in ZUFSP. The distal ubiquitin is stabilised by extensive contacts with the ZHA domain and the catalytic core. The MIU motif is partially ordered and does not mediate any interactions with the distal ubiquitin. Ubiquitin binding to MIU was modelled based on the canonical mode of MIU-Ub interaction (PDB ID: 2FIF). Superposition of the RABEX5 MIU-Ub interaction onto the MIU motif of ZUFSP results in ubiquitin binding to the MIU motif in such a way that the C-terminus of the MIU-bound ubiquitin points towards K63 of the ZHA bound distal ubiquitin. This model therefore suggests a K63-linked diubiquitin bound to the long helical arm, i.e. ZHA and MIU. Further, it suggests that the MIU may serve as an S2 binding site. Indeed, mutation of the S2 binding site results

in slightly reduced DUB activity (**Figure S5F**). The polyubiquitin binding UBZ domain (ZNF4) is required for catalytic activity and we suggest that it may form the proximal ubiquitin binding site.

**Supplementary Tables (related to Figure 1, 2, 3, 4 and 5).**

Table S1

All DNA constructs were made in backbone of the pGEX6P vector for GST-tagged protein unless indicated otherwise.

| PROTEIN | DU NUMBER | CONSTRUCT BOUNDRIES |
|---|---|---|
| INTEIN constructs | | |
| HA-Ufm1* | 47655 | 1-82 |
| HA-UB* | 47186 | 1-82 |
| HALO-C3-Ub* | 55054 | 1-75 |
| DUBs | | |
| Ufsp1 (Mm) | 55016 | 1-448 |
| OTUD6A** | 20889 | 1-288 |
| ZUFSP HALO-tag constructs | | |
| ZUFSP HALO-MIU | 55532 | 225-267 |
| ZUFSP HALO-ZNF3,4,MIU | 24215 | 145-267 |
| ZUFSP HALO-ZNF4-MIU | 55605 | 186-578 |
| ZUFSP HALO-ZNF3,4 | 55606 | 147-220 |
| ZUFSP HALO-ZNF3 | 55703 | 147-186 |
| ZUFSP HALO-ZNF4 | 55689 | 186-225 |
| ZUFSP HALO-ZNF1,2 | 55762 | 1-66 |
| ZUFSP HALO-ZNF1 | 55764 | 1-24 |
| ZUFSP HALO-ZNF2 | 55786 | 26-66 |
| ZUFSP HALO-ZNF1-4,ZHA,MIU | 55787 | 1-267 |
| ZUFSP HALO-ZNF1-4 | 55763 | 1-217 |
| ZUFSP HALO-ZNF4 L200A, I201A | 55955 | 186-225 |
| ZUFSP HALO-ZNF4 N204A, Y205A | 55956 | 186-225 |
| ZUFSP HALO-ZNF4 I207A, L208A | 55957 | 186-225 |
| ZUFSP HALO-ZNF4 Q209A, E210A | 55958 | 186-225 |
| DUB assay constructs | | |
| $ZUFSP^{Cat}$ | 53622 | P294-578 |
| $ZUFSP^{ZHA\text{-}Cat}$ | 27537 | 248-578 |
| $ZUFSP^{MIU\text{-}Cat}$ | 53621 | S231-578 |

| | | |
|---|---|---|
| $ZUFSP^{ZNF4\text{-}MIU\text{-}Cat}$ | 56330 | 184-578 |
| $ZUFSP^{ZNF3,4,MIU\text{-}Cat}$ | 55685 | S145-578 |
| $ZUFSP^{ZNF4\text{-}MIU\text{-}Cat}$ C360A | 55760 | 184-578 |
| $ZUFSP^{ZNF4\text{-}MIU\text{-}Cat}$ C360S | 59209 | 184-578 |
| $ZUFSP^{ZNF4\text{-}MIU\text{-}Cat}$ H491A | 55797 | 184-579 |
| $ZUFSP^{ZNF4\text{-}MIU\text{-}Cat}$ Y267A | 59010 | 184-579 |
| $ZUFSP^{ZNF4\text{-}MIU\text{-}Cat}$ L269A | 59011 | 184-579 |
| $ZUFSP^{ZNF4\text{-}MIU\text{-}Cat}$ Q487A | 59040 | 184-579 |
| $ZUFSP^{ZNF4\text{-}MIU\text{-}Cat}$ D512A | 59064 | 184-579 |
| $ZUFSP^{ZNF4\text{-}MIU\text{-}Cat\ L236A}$ A237G | 59168 | 184-579 |
| $ZUFSP^{ZNF4\text{-}MIU\text{-}Cat\ I207A,}$ L208A | 59189 | 184-579 |
| $ZUFSP^{MIU\text{-}Cat\text{-}linker\text{-}ZNF4}$ | 55967 | 250-578-5XGS-186-225 |

*pTXB1

**pET156P-1

***pMEX3C
